# Comprehensive Structural Characterization of the Bacterial Homospermidine Synthase–an Essential Enzyme of the Polyamine Metabolism

**DOI:** 10.1038/srep19501

**Published:** 2016-01-18

**Authors:** Sebastian Krossa, Annette Faust, Dietrich Ober, Axel J. Scheidig

**Affiliations:** 1 Structural Biology–Zoological Institute, Kiel University, Am Botanischen Garten 11, 24118 Kiel, Germany; 2 Botanical Institute – Biochemical Ecology and Molecular Evolution, Kiel University, Am Botanischen Garten 1-9, 24118 Kiel, Germany

## Abstract

The highly conserved bacterial homospermidine synthase (HSS) is a key enzyme of the polyamine metabolism of many proteobacteria including pathogenic strains such as *Legionella pneumophila* and *Pseudomonas aeruginosa*; The unique usage of NAD(H) as a prosthetic group is a common feature of bacterial HSS, eukaryotic HSS and deoxyhypusine synthase (DHS). The structure of the bacterial enzyme does not possess a lysine residue in the active center and thus does not form an enzyme-substrate Schiff base intermediate as observed for the DHS. In contrast to the DHS the active site is not formed by the interface of two subunits but resides within one subunit of the bacterial HSS. Crystal structures of *Blastochloris viridis* HSS (*Bv*HSS) reveal two distinct substrate binding sites, one of which is highly specific for putrescine. *Bv*HSS features a side pocket in the direct vicinity of the active site formed by conserved amino acids and a potential substrate discrimination, guiding, and sensing mechanism. The proposed reaction steps for the catalysis of *Bv*HSS emphasize cation-π interaction through a conserved Trp residue as a key stabilizer of high energetic transition states.

Polyamines are essential for cell functioning, growth, proliferation and for apoptosis and are found in nearly all living species^1^,^2^,^3^. The prominent triamines (Fig. 1b) in bacteria are *sym*-homospermidine (bis(4-aminobutyl)amine, HSP), spermidine ((4-aminobutyl)(3-aminopropyl)amin, SPD), and *sym*-norspermidine^2^. Eukaryotes generally utilize SPD or spermine of which SPD is the precursor of hypusine, an amino acid resulting from the post-translational modification of the elongation factor eIF-5A^1^. Activated eIF5A is essential for eukaryotes as it is involved in translation elongation^4^. The first step of the post-translational activation of eIF5A is catalyzed by deoxyhypusine synthase (DHS, EC 2.5.1.46) that transfers the amino butyl moiety of SPD to a specific lysine residue of the eIF5A precursor protein. As a side activity, DHS catalyzes also the formation of HSP by using putrescine (1,4-diaminobutanal, PUT) as acceptor^5^. This activity was selected and optimized after duplication of the *dhs*-encoding gene several times independently during angiosperm evolution, resulting in homospermidine synthase (HSS, EC: 2.5.1.45)^6^,^7^. HSS is the first pathway-specific enzyme of pyrrolizidine alkaloid biosynthesis, a class of toxic compounds involved in the plant’s chemical defense. A distinct feature of DHS and HSS is the use of NAD^+^as a prosthetic group, which functions as a hydride acceptor and donor during the two step reaction^8^. They have this feature in common with a bacterial enzyme that catalyzes an apparently almost identical reaction, i.e. bacterial HSS (EC: 2.5.1.44). As with DHS and HSS of eukaryotic origin, bacterial HSS has been found to be active at neutral to basic pH with an optimum pH range of 8.7 to 9^9^,^10^. In contrast to its eukaryotic counterparts, bacterial HSS synthesizes 1 mol HSP from 2 mol PUT (see Fig. 1)^9^. In contrast, eukaryotic DHS and HSS are unable to synthesize HSP from PUT only. They only accept SPD and HSP as donor for the aminobutyl moiety^11^. It was shown for eukaryotic DHS that a Schiff base is formed during the transfer of the aminobutyl moiety, an intermediate also suggested to be involved in the reaction of bacterial HSS^12^.

The bacterial HSS is highly conserved and is proposed to be evolutionarily related to carboxy(nor)spermidine dehydrogenase (CA(N)SDH, EC: 1.5.1.43)^13^. CA(N)SDH together with carboxy(nor)spermidine decarboxylase (CA(N)SDC, EC: 4.1.1.96) are essential enzymes of an alternative SPD biosynthetic pathway utilized by many bacteria not possessing HSP^14^. HSS from some but not all species are potassium-dependent with an optimum of, for example, 50 mM for *Blastochloris viridis* HSS (*Bv*HSS)^9^,^10^. The enzyme is capable of catalyzing side reactions to produce a variety of N-aminobutyl-linked triamines utilizing PUT together with respective linear diamines with C3 to C7 carbon chains (see Fig. 1)^9^. The bacterial HSS can produce HSP from SPD as its sole substrate^8^. 1,3-Diaminopropane (DAP) is the only known diamine functioning as a strong competitive inhibitor of bacterial HSS^9^,^10^,^12^. The activity of bacterial HSS in producing HSP is slightly reduced under the administration of PUT together with either 1-aminopropane, 1-aminobutane, 1,2-diaminoethane, 1,5-diaminopentane (CAD), 1,6-diaminohexane, and 1,7-diaminoheptane or SPD^9^,^10^.

The bacterial HSS is present in many α-, γ-, and δ-proteobacteria and, in particular, in pathogenic strains such as *Legionella pneumophila, Brucella* spp., and *Pseudomonas aeruginosa*^13^. In addition, the bacterial HSS seems to be present in some, mainly unicellular, eukaryotes^13^,^15^. Increasing numbers of multidrug resistant pathogens in general raise the need for new antibiotic targets^16^. The essential function of HSP for growth, in addition to the difference in the mechanism and evolution of its synthesizing enzymes in bacteria and eukaryotes, suggests that bacterial HSS represents such a potential target^6^,^13^. Furthermore, polyamines are crucial in bacteria-host interactions and are involved in bacterial pathogenesis, growth rate, biofilm formation and they activate mechanisms for the evasion or repression of the host immune system^3^,^17^,^18^,^19^.

Studying the evolution of plant HSS from eukaryotic DHS, we were interested whether there might be an evolutionary link to the bacterial HSS. Despite of the low amino acid sequence identity between plant HSS and bacterial HSS of about 12% (*Senecio vulgaris* vs. *Blastochloris viridis* HSS), a conserved fold within the three dimensional structure of bacterial HSS might be responsible for the similarity of the reaction mechanism. As the structure of human DHS is available in the databases and its sequence is highly identical to that of other eukaryotic DHS and HSS, we decided to analyze the three dimensional (3D) structure of the bacterial HSS of *Blastochloris viridis* in more detail. *Blastochloris viridis* is a gram-negative α-proteobacteria. This organism is a photoheterotroph harboring one of the simplest photosynthetic systems and is frequently used as a model system for studying plant biochemistry and pathways^20^. Our study will lead to a better understanding of the enzyme function and elucidates potential similarities or differences to eukaryotic HSS/DHS. In addition, these insights are mandatory to assess the potential of the bacterial HSS as an antibiotic drug target. Here, we present high resolution structures of *Bv*HSS and variants with various bound polyamines, providing a detailed insight into substrate-binding properties and function of bacterial HSS.

## Results and Discussion

### Overall Structure of *Blastochloris viridis* HSS

The *Bv*HSS structure was solved from crystals belonging to space group P2_1_2_1_2_1_ with bound NAD^+^ (Protein Data Bank [PDB] ID: 4PLP). Crystals from *Bv*HSS and *Bv*HSS variants with bound NAD^+^ in complex with various polyamines all belonged to space group P22_1_2_1_ with cell parameters in approximately the same order of magnitude. Detailed data collection statistics are summarized in Table 1.

**Figure 2:**
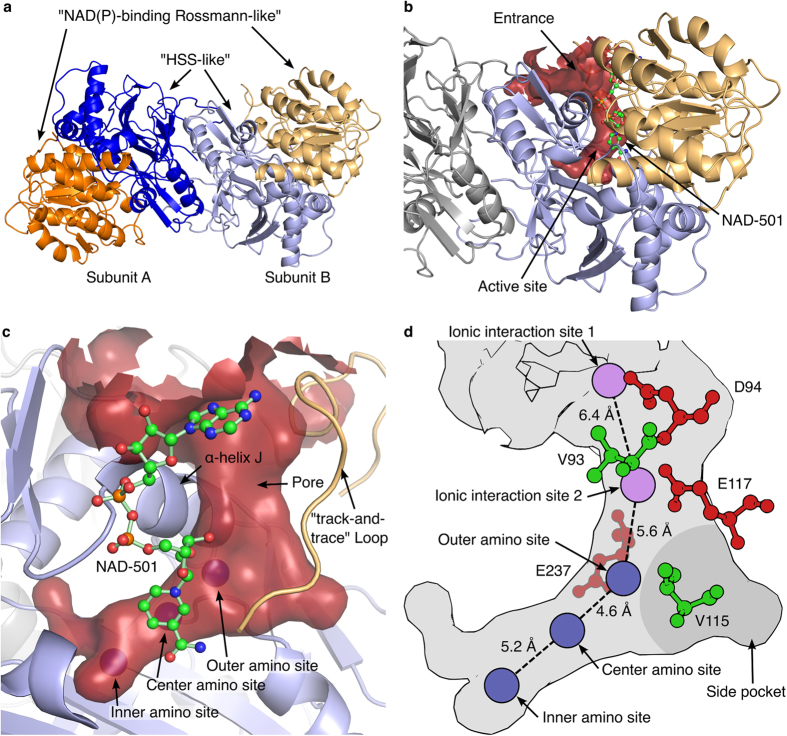
Overall structure of the homodimeric *Bv*HSS. (**a**) Cartoon representation of the *Bv*HSS (PDB ID: 4PLP) dimer with domain 1 (“NAD(P)-binding Rossmann-like”) in orange (subunit A)/light orange (subunit B) and domain 2 (“homospermidine-synthase (HSS)-like”) in blue (subunit A)/light blue (subunit B). (**b,c**) Substrate binding pocket displayed as a surface-rendered cavity in red with adjacently bound NAD^+^ (in ball-and-stick representation). (**c**) Outer, center, and inner amino sites are indicated as blue spheres inside the red surface-rendered cavity, and the “track-and-trace” loop is given as a cartoon representation in light orange. (**d**) Representation of the substrate-binding pocket shown in (**c**). Amino sites are displayed as blue circles, and ionic interaction sites are displayed as violet circles. Amino acids providing the “ionic slide” (ball-and-stick representation) with acidic side chains are colored in red, and those with non-polar side chains are colored in green. The approximate position of the side pocket on the rear side of the binding pocket is indicated as a dark gray area.

The crystal structure of *Bv*HSS was identified as a dimer with bound NAD^+^ (PDB entry 4PLP) in the asymmetric unit (Fig. 2a). The first four amino acids could not be traced for each of the two protein subunits. In each subunit, twelve residues (2% of residues per subunit) were only poorly represented by electron density as indicated by residue type and resolution normalized real space R-values (RSRZ) given in the worldwide PDB (wwPDB) structure validation report^21^,^22^. All related residues were located at the protein surface within loop regions. The interface area of the dimer was calculated as approximately 1700 Å^2^ by PDBsum indicating a stable dimer^23^. The *Bv*HSS subunit consists of two domains as determined by CATH^24^: one “NAD(P)-binding Rossmann-like” domain (domain 1, residues 3–163 and 394–425) and one “homospermidine-synthase-like domain” (domain 2, residues 164–395 and 426–477) as shown in Fig. 2a and S1. Both subunits are structurally very similar with a root-mean-square distance (RMSD) of 0.8 Å^2^ (for details see Table 2). Subsequently, determined structures of *Bv*HSS and *Bv*HSS variants with bound NAD^+^ and different bound polyamines (compare Table 3) were found to be highly similar to the *Bv*HSS structure with PDB ID: 4PLP (RMSDs in Table 4). Slightly different relative orientations of subunit A to subunit B were observed for all *Bv*HSS structures. The most prominent differences were ascertained for a major fraction (residues Leu-120 to Pro-130) of a loop region (residues Thr-114 to Pro-130, further referred to herein as “track-and-trace” loop, see also Fig. 2c and S1) between the holoenzyme (PDB ID: 4PLP) and the substrate (PUT/HSP)-bound form (PDB ID: 4TVB). Alignment of HSS from *L. pneumophila* subunit A (PDB ID: 2PH5) with *Bv*HSS subunit A (PDB ID: 4PLP) resulted in an RMSD of 1.1 Å^2^ (362 to 362 C_α_ atoms, after 5 cycles of the PyMOLs “super” algorithm) indicating similar 3D structures as expected from the sequence alignment.

The bacterial HSS is supposed to be evolutionarily related to CA(N)SDH, lysine 6-dehydrogenase, saccharopine dehydrogenase, and aspartate dehydrogenase^13^. This relationship is supported by the superposition of a bacterial saccharopine dehydrogenase from *Wolinella succinogenes* (EC: 1.5.1.7, PDB ID: 4INA, RMSD 3.2 Å^2^, 224 to 224 C_α_ atoms, as described above) and two eukaryotic saccharopine dehydrogenases/reductases from *Saccharomyces cerevisiae* and *Magnaporthe grisea* (EC: 1.5.1.10, PDB ID: 2AXQ^25^ with RMSD 3.6 Å^2^, 127 to 127 C_α_ atoms and PDB ID: 1E5Q^26^ with RMSD 2.9 Å^2^, 116 to 116 C_α_ atoms, as described above) with *Bv*HSS (PDB ID: 4PLP) subunit A indicating a similar overall fold.

The crystal structures of *Bv*HSS containing NAD^+^ clearly show that the active site is localized inside each subunit. It is not formed by the interface region of the dimer as observed for homotetrameric DHS^27^.

### NAD(H) Binding Site

NAD^+^ serves as a non-covalently bound prosthetic group for *Bv*HSS. It is coordinated through hydrogen bonding via residues Ser-21, Ile-22, Ser-230 (phosphate), Asp-45, Val-66 (adenosine), Ser-92, Thr-114, Ala-161, Asn-162, and Pro-163 (nicotineamide riboside). The phosphate-binding motif (^18^GFGSIG^23^) is located in the loop connecting β-strand 2 and α-helix A of the Rossmann fold. The adenosine part of NAD^+^ is bound via loop regions located between β-strand 4, 5, 6 and α-helix C, D, E. Nicotineamide-riboside-binding residues are found in loop regions between β-strand 7 and 8 and α-helix F and O.

### Characteristics of the Binding Pocket of *Bv*HSS

**Figure 3:**
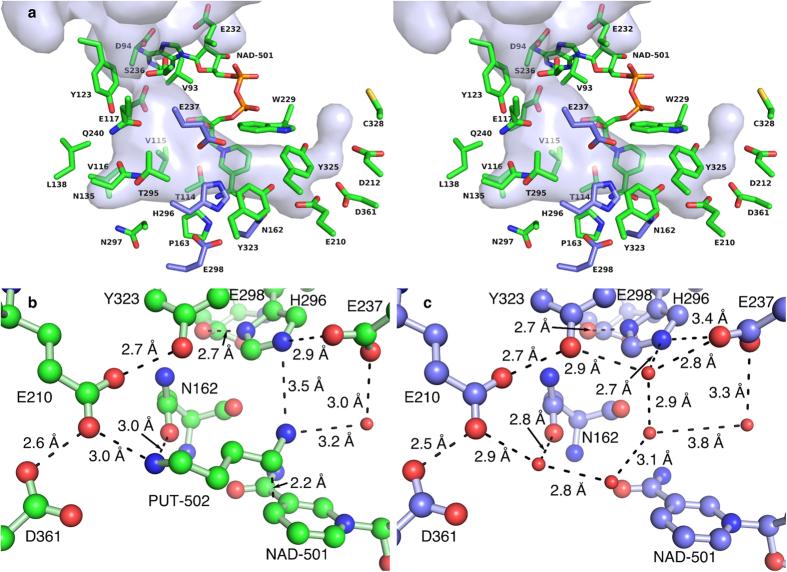
Residues of the binding pocket of *Bv*HSS. (**a**) Walled-eye 3D stereoscopic visualization of all relevant residues near the binding pocket of *Bv*HSS. The substrate binding pocket is displayed as a surface-rendered cavity in light blue with the adjacently bound NAD^+^ and the side chains including the C_α_ carbons of the relevant residues (in stick representation). The carbons of residues replaced in the four variants are colored in blue, all other carbons are colored in green. (**b,c**) Relevant residues, NAD-501, and PUT-502 at the active site are shown as ball-and-stick representations. Relevant water molecules are shown as red spheres. All distances (indicated by dashed lines) were measured with the measurement function implemented in the program PyMOL. To avoid the mis-interpretation of panel (**b,c**) as a stereo representation, the carbons are shown in green (**b**) or blue (**c**). (**b**) The active site of *Bv*HSS with bound PUT (PDB ID: 4TVB, subunit B). (**c**) The active site of *Bv*HSS without substrate (PDB ID: 4XQC, subunit B).

**Figure 4:**
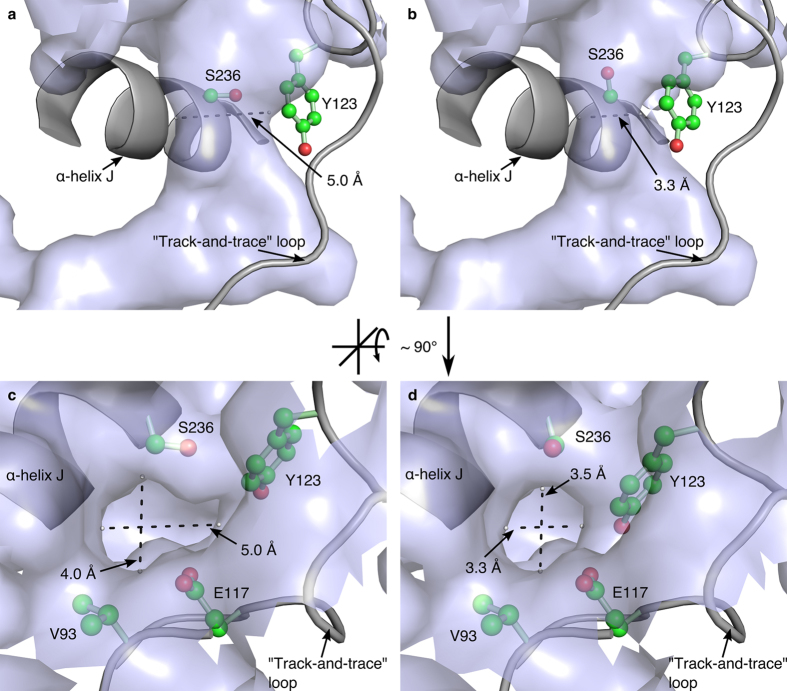
Dimensions of the pore at the entrance of the binding pocket. The binding pocket of *Bv*HSS variant H296S, subunit A (PDB ID: 4XQE) is represented as a surface-rendered cavity in light blue. Pore-forming α-helix J and the “track-and-trace” loop are shown as cartoon representations. Pore-forming amino acids (Val-93, Glu-117, Tyr-123, and Ser-236) are given in ball-and-stick representations. The two observed alternate conformations A (**a,c**) and B (**b,d**) of residues Tyr-123 and Ser-236 effecting the dimension of the pore are shown separately. All given dimensions of the pore for both orientations and alternate conformations were measured in the same plane. All distance measurements were performed with the measurement function implemented in the program PyMOL.

The volume and the surface of the binding pocket were calculated based on artificial water molecule coordinates generated with the software HOLLOW^28^ by filling the interior of the protein with dummy atoms (1.4 Å radius) on a grid (spacing 0.2 Å). Water molecules of the respective crystal structure of *Bv*HSS present inside or in the direct vicinity of the generated volume were manually added to the artificial water molecules prior to surface calculation. The pocket is a “boot-shaped” cavity that is approximately 21 Å deep (Figs 2d and 3a). The entry to the pocket is formed by part of the “track-and-trace” loop (residues Phe-122 to Asp-125) and the α-helix J at the protein surface (Fig. 2b,c). The innermost end lies near amino acids Asn-162 and Glu-210. The active site is situated between residues Asn-162, Trp-229, Glu-237, His-296, and the nicotine amide ring of NAD^+^ at the narrow end of the pocket (Fig. 3). Residues Val-115, Val-116, Tyr-123, Asn-135, Leu-138, Pro-163, Gln-240, Thr-295, and Asn-297 form a side pocket at the “heel” of the “boot-shaped” binding pocket (Figs 2d and 3a), which is filled with six ordered water molecules of which five occur in a nearly planar and equidistant five-membered ring. At least five amino acids (Val-93, Glu-117, Tyr-123, Gly-233, and Ser-236) form a pore at the binding pocket entrance. Based on these observations, four variants of *Bv*HSS (N162D, E237Q, H296S, and E298Q) have been generated for further characterization of residues within the active site (Fig. 3a). All side chains of these functionally relevant residues (with the exception of Glu-298) participate in forming the active site (Fig. 3). Residue Glu-298 has been chosen based on direct interaction with the active site residue His-296, forming a Glu-His-Glu triad together with Glu-237 (Fig. 3). All four variants of *Bv*HSS lead to enzymatically inactive but soluble enzymes. The structure of the *Bv*HSS variant H296S (PDB ID: 4XQE) has revealed two alternative conformations for the “track-and-trace” loop and for Ser-236 (α-helix J, Fig. 4). These alternative conformations result in at least two different possible dimensions of the pore opening. Narrowing might occur by the bending of the “track-and-trace” loop resulting in a slight rotation of the side chain of Tyr-123 deeper into the pocket and towards Glu-117, accompanied by a side chain rotation of Ser-236 towards the pocket entrance. Thus, the ellipsoid-shaped pore is narrowed from an approximately 4.0 Å times 5.0 Å opening to an approximately 3.3 Å times 3.5 Å opening (Fig. 4). Thus, a hypothetical locking or a substrate sensing and discriminating mechanism at the binding pocket entrance could sense changes in substrate binding via the “track-and-trace” loop. It runs mainly alongside the binding pocket and could directly interact with the nicotine amide ring of NAD^+^ through Thr-114. In particular, side chains of residues Val-115, Glu-117, and Tyr-123 reaching into the binding pocket provide an alternating polar/apolar path. Additional sensing might occur through Glu-237, which is adjacent to the pore-forming Ser-236. Its side chain reaches directly into the active side and interacts with residue His-296.

**Figure 5:**
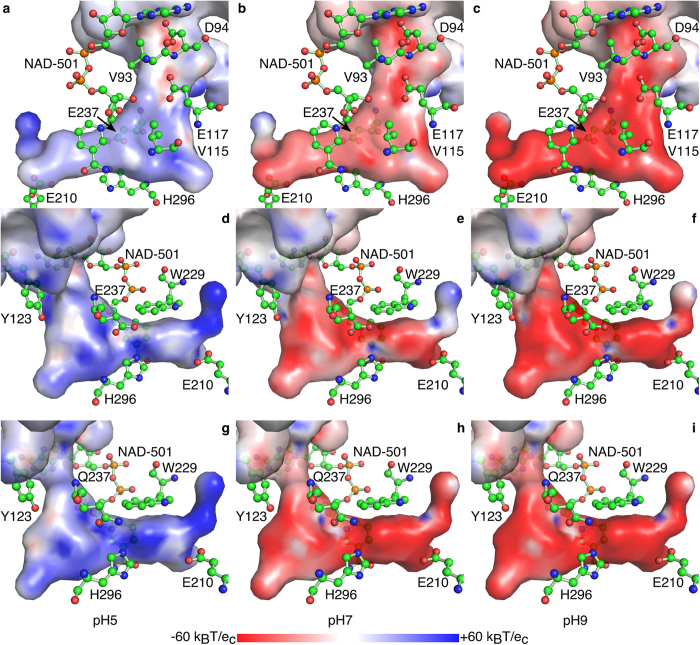
Representation of the electrostatic potential of the surface of the binding pocket at pH 5, 7, and 9. The electrostatic potential at the surface of the substrate-binding pocket (compare with Fig. 2b,c) is represented as a color gradient from red (−60 k_B_T/e_c_) over white (0 k_B_T/e_c_) to blue (+60 k_B_T/e_c_) at pH 5 (**a,d,g**), pH 7 (**b,e,h**), and pH 9 (**c,f,i**) from two opposing orientations for *Bv*HSS wild-type (a-f, PDB ID: 4PLP) and one orientation for *Bv*HSS variant E237Q (g-i, PDB ID: 4XQG). The cofactor NAD^+^ and the important residues Val-93, Asp-94, Val-115, Glu-117 (“ionic slide”), Tyr-123, Glu-210, Trp-229, Glu-237, and His-296 are superimposed in ball-and-stick representation. Residues Glu-232, Glu-298, Tyr-323, and Tyr-325 are not shown for clarity, see Fig. 3 instead.

Under the assumption that the coordinates of the enzyme do not significantly change upon variation of the pH-value of the bulk solvent, the electrostatic properties of the binding pocket were analyzed by calculating the side chain pK_a_-values based on the 3D structure of *Bv*HSS (PBD # 4PLP) and subsequently derived electrostatic potential maps at pH 5, 7, and 9 (visualized in Fig. 5).

The resulting overall electrostatic potential of the surface of the pocket is mainly negative at pH 7 and pH 9, in contrast to a mainly positive potential at pH 5 (compare Fig. 5b,c,e,f with Fig. 5a,d). At pH 7 (or pH 9), the negative electrostatic potential at the surface of the pocket increases from a region immediately above the entrance down to the active site. The entrance and active site are conjoined by a path along the surface of the binding pocket with areas of highly negative electrostatic potential (mainly provided by Asp-94, Glu-117, and Glu-237 from entrance to active site). The residue His-296 of the Glu-His-Glu triad with a calculated pK_a_ value of 10 is most probably protonated and positively charged at both side chain nitrogens at pH 5, pH 7, and pH 9 most likely causing the only positive electrostatic surface potential at pH 7 and pH 9 in the direct vicinity of the center amino site where the hydrid transfer between the prosthetic group NAD(H) and the substrate takes place. The reported catalytic pH optimum of pH 9 for HSS^9^ is in agreement with the calculated electrostatic potential of the surface of the binding pocket. PUT and HSP or other di- and tri-amines with a theoretical pK_a_ value of 10 for their terminal amino groups will mainly be positively charged at pH 5 to pH 9. The binding pocket appears to facilitate, but only under neutral to basic conditions, a mainly negative electrostatic surface potential and thus an attractive electrostatic effect on its substrates, thus explaining the observed pH-optimum of pH 8.7-9 for bacterial HSS^9^,^10^. A strongly negative electrostatic surface potential at the pore entrance might function as “bait” for the positively charged substrate, which then “slides down” the negative potential towards the active site. In particular, the side chain carboxy groups of Asp-94, Glu-117, and Glu-237 are positioned such as to provide negative ionic interaction sites at a distance of approximately 6.4 Å and 5.6 Å, with a small hydrophobic site (Val-93 or Val-115) in between each (Fig. 2d). With regard to the nitrogen-nitrogen distance of approximately 6.1 Å of linear PUT and the highly conserved residues Glu-117 and Glu-237^13^, this arrangement (“ionic slide”) might be involved in substrate discrimination and direction towards the active site. Because of its positive electrostatic potential, the edge of the aromatic side chain of Tyr-123 (being part of the track-and-trace loop) might additionally support substrate transfer by “pushing it down” by electrostatic repulsion.

In contrast to the bacterial HSS, the active site of the human DHS is present at the interface of two subunits of the homotetramer forming a rather straight and approximately 17 Å deep tunnel^29^. In addition, the most significant difference is the absence of a lysine residue at the active site of the bacterial HSS as utilized by the DHS to transiently store the reaction intermediate 4-aminobutanal^29^,^30^. The residues Asp-238, His-288, and Asp-316 of the human DHS, all of functional relevance^31^, are arranged in a comparable triad like that of Glu-237, His-296, and Glu-298 in bacterial HSS. Like the bacterial HSS, the human DHS contains a functional relevant tryptophane (Trp-327) near the hydrid transfer site^29^,^31^. Besides a mostly negative electrostatic surface potential no further similarities between both active sites exist underlining the different evolutionary origins of both enzymes. There has been no structure of an eukaryontic HSS reported yet. Nevertheless, homology models, evolutionary origin and resemblance of catalyzed reactions suggest similar structural characteristics of the active site as for the DHS^6^,^29^.

### Substrate-binding Sites of *Bv*HSS

To determine substrate-binding sites, *Bv*HSS and the non-functional *Bv*HSS variants H296S and E237Q were co-crystallized or soaked with diamines of different lengths (DAP, PUT, and CAD) and with the polyamine biosynthesis inhibitor agmatine (AGM) under various conditions as given in Table 3.

### HPLC Analysis of *Bv*HSS Crystals Supports Electron Density Interpretation

The type of polyamine present within the individual crystals was verified by HPLC analysis. The analyses of a *Bv*HSS crystal cluster co-crystallized with DAP and PUT (sample A) and of a *Bv*HSS crystal cluster (sample B) and a single *Bv*HSS crystal (sample C), both co-crystallized with DAP and soaked with PUT, clearly detected DAP and PUT plus SPD in all samples. The height and area of the DAP peaks detected in samples B and C were lower than that of the PUT peak, in contrast to an approximately equal height and area detected in sample A (chromatograms shown in Fig. S2 and S3). This indicates an effective reduction of compounds present in the crystallization solution by the crystal transfer and polyamine derivatization procedure and thereby emphasizes the polyamine composition inside the *Bv*HSS crystals. All analyzed crystals gave a peak with the same retention time of HSP; this peak was overlaid by a peak of an unknown impurity or side product of the labeling procedure. The peak height and area of this impurity detected during calibration runs without HSP was lower than the peak detected during the analysis of the *Bv*HSS sample A and was approximately of the same or slightly higher magnitude as that during the analysis of sample C (Fig. S2a vs. d and S3 a vs. b, d). The presence of SPD (known *Bv*HSS product of DAP and PUT^9^) in all samples and the most probable presence of HSP in sample A, together with the effective reduction of mother liquor compounds, strongly indicate that *Bv*HSS is active under these crystallization conditions.

### *Bv*HSS Soaked with PUT Contains Transition-close States of the Catalyzed Reaction

**Figure 6:**
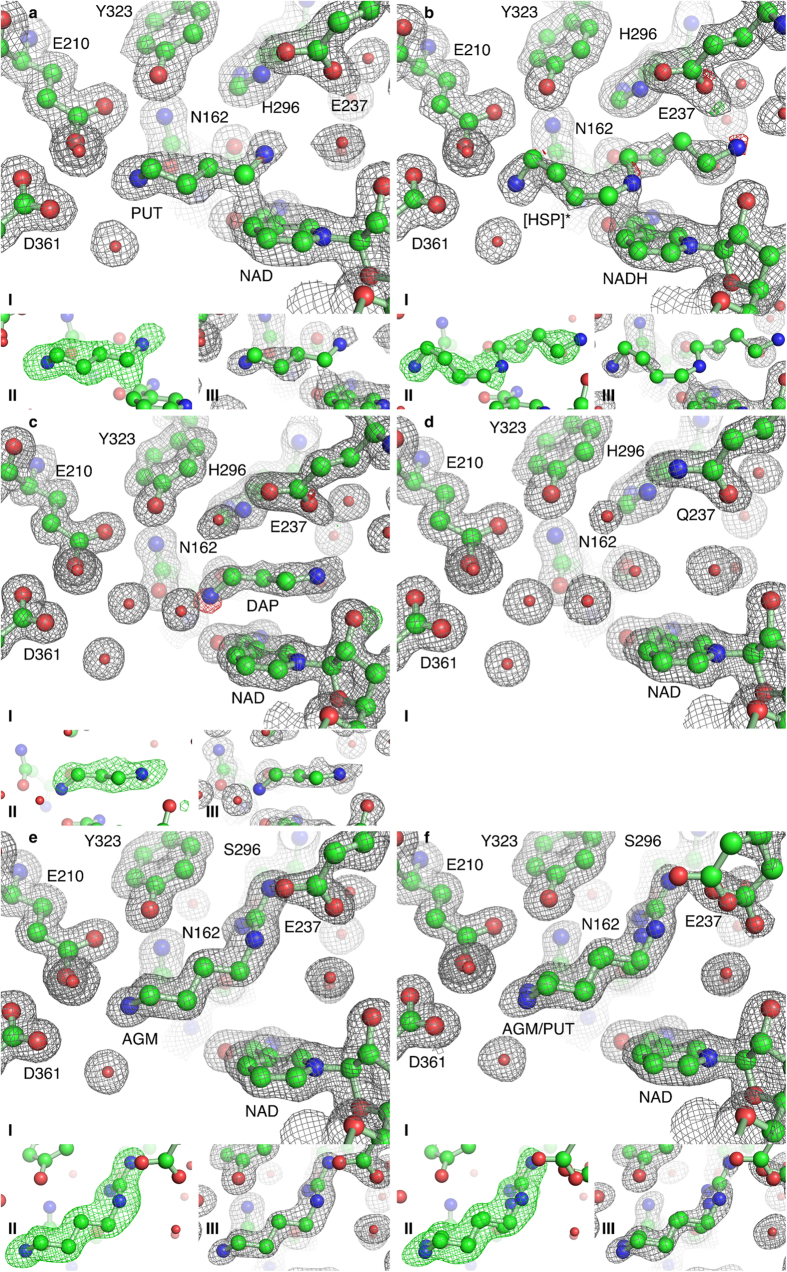
Active site of *Bv*HSS and *Bv*HSS variants E237Q and H296S with bound polyamines. The electron density maps are shown as a mesh at a contour level of 1σ (2mFo-DFc in gray) and +/− 5σ (mFo-DFc, green/red). Electron density maps obtained from PHENIX.refine are shown in (I), and the simulated annealing (SA) ligand omit electron density (ED) maps obtained from PHENIX.composite_omit_map are shown in (II, mFo-DFc) and (III, 2mFo-DFc). Relevant residues are shown as ball-and-stick representations. (**a,b**) Active site of *Bv*HSS (PDB ID: 4TVB) with bound substrates in subunit A and subunit B. The PUT bound in subunit B is shown in (**a**). The transition close state of the reduction to HSP bound in subunit A is shown in (**b**). (**c**) Active site of *Bv*HSS with bound DAP (subunit A, PDB ID: 4XQE). (**d**) Active site of *Bv*HSS variant E237Q (subunit B, no SA ligand omit ED maps were calculated because of the lack of bound substrate, PDB ID: 4XQG). (**e,f**) Active site of *Bv*HSS variant H296S with bound AGM (e, PDB ID: 4XQE, subunit B) or with bound AGM and PUT as alternate conformations (f, PDB ID: 4XRG, subunit B). The adjustment of the occupancy values for AGM and PUT (with a constrained occupancy group per molecule to ensure equal occupancy for each atom of the respective molecule) was performed by PHENIX.refine during refinement. The calculated occupancy is in subunit A 0.56 for AGM and 0.44 for PUT and in subunit B 0.66 for AGM and 0.34 for PUT.

**Figure 7:**
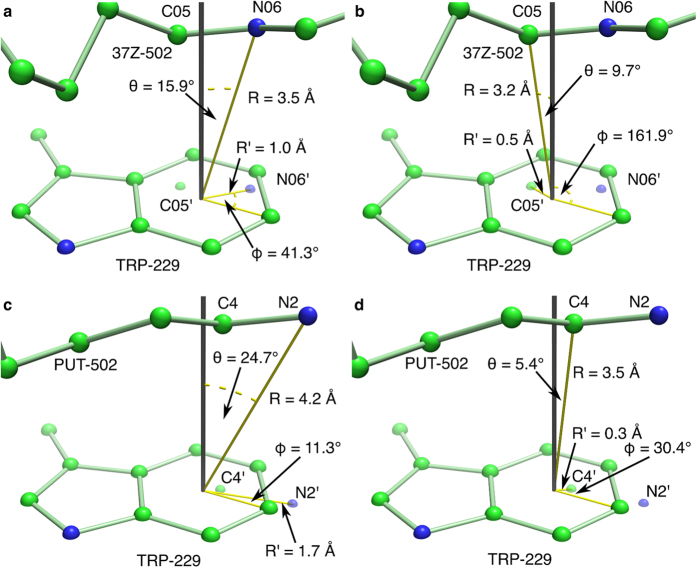
Tryptophan Trp-229 cation-π interaction angles and distances. The *Bv*HSS (PDB ID: 4TVB) subunit A Trp-229 and HSP (residue 37Z-502 in PDB ID: 4TVB) (**a,b**) and subunit B Trp-229 and PUT-502 (**c,d**) are shown in ball-and-stick representations. The plane of the indol ring of the Trp-229 side chain is indicated by a white surface cutting all spheres in half representing atoms lying on the aromatic plane. The black line represents the orthogonal vector to the aromatic plane with its origin at the center of the 6-membered (benzene) ring of the tryptophan side chain. The orthogonal projections of carbon C05 and nitrogen N06 of 37Z-502 (**a,b**) and of carbon C4 and nitrogen N2 of PUT-502 (**c,d**) are indicated as slightly smaller spheres named C05′, N06′, C4′, and N2′. All angle and distance measurements were performed with the measurement function implemented in the program PyMOL. The names of the angles and distances were chosen by analogy to Marshall *et al*.^32^.

Supported by the results from the HPLC analyses, the electron density derived from a *Bv*HSS crystal co-crystallized with DAP and subsequently soaked for 5 min with PUT was interpreted to contain “transition-close” states of the catalyzed reaction (PDB ID: 4TVB, Fig. 6a,b). The electron density at the active site of subunit B is interpreted as a hydride transfer from PUT carbon C4 to NAD^+^ carbon C4N representing a “transition-close” state of the oxidation at carbon C4. The electron density at the active site of subunit A is interpreted as representing the hydride transfer from carbon C4N of NADH to the still oxidized carbon C05 of HSP, with a partial double bond between carbon C05 and nitrogen N06 of HSP representing a partial Schiff base (see subsection “Hypothesis of the Reaction Steps of *Bv*HSS Catalysis” for details). The inner most amino group at nitrogen N01 of HSP or nitrogen N1 of PUT is coordinated to residues Glu-210 and Asn-162 (inner amino site), whereas the amino group at nitrogen N11 of HSP is coordinated to Glu-237 and oxygen O2D of NADH (outer amino site). The nitrogen N2 of PUT is coordinated to Asn-162 (center amino site). The polyamine nitrogens (N2 of PUT, N06 of HSP) are bound with a non-optimal off-center geometry relative to the center of the 6-membered (benzene) ring of the respective Trp-229 side chain (Fig. 7a,c). Based on calculated interaction energies of benzene with NH_4_^+^^32^, these positions will most likely result in interaction energies significantly contributing to substrate binding and recognition via cation-π interaction^33^. The substrates are further stabilized by hydrophobic interactions with Val-115, Pro-163, Trp-229, His-296, Tyr-323, Tyr-325, and Thr-396. The binding position between the inner amino site and center amino site will be referred to as the inner (binding) site, whereas the binding position between the center amino site and outer amino site will be referred to as the outer (binding) site (Fig. 2).

### DAP Binds at the Active Site and at the Ionic Slide

**Figure 8:**
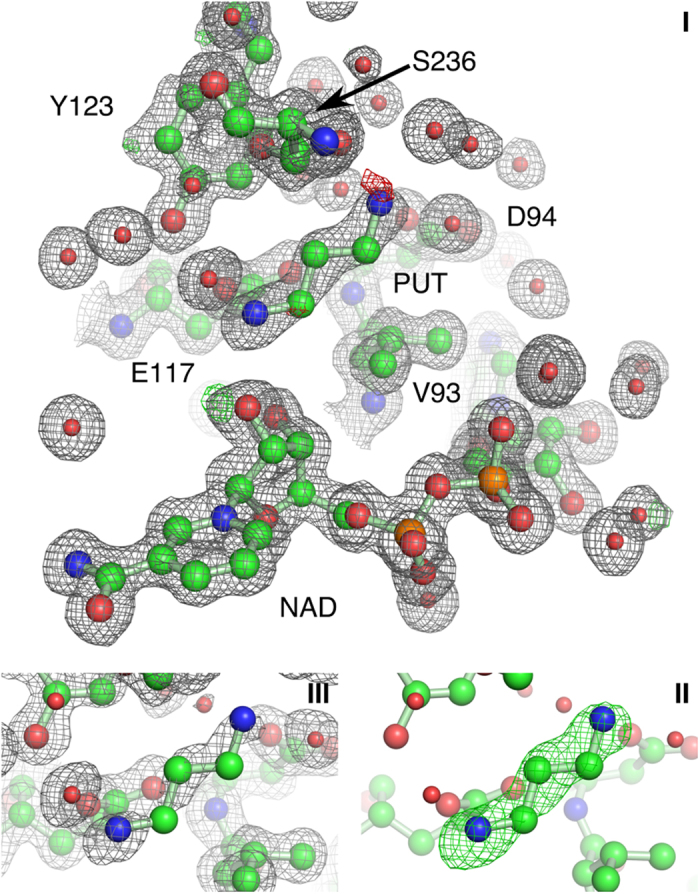
DAP bound at the “ionic slide” of *Bv*HSS (PDB ID: 4XQC). The electron density map of relevant residues, NAD^+^ and DAP are shown as a mesh at a contour level of 1σ (2mFo-DFc in gray) and +/− 5σ (mFo-DFc, green/red). Electron density maps obtained from PHENIX.refine are shown in (I), and the simulated annealing (SA) ligand omit electron density (ED) maps obtained from PHENIX.composite_omit_map are shown in (II, mFo-DFc) and (III, 2mFo-DFc). Relevant residues are shown as ball-and-stick representations.

The electron density obtained from a *Bv*HSS crystal co-crystallized with DAP and PUT (PDB ID: 4XQC) did not allow an interpretation of *Bv*HSS with bound PUT or HSP, but rather with bound DAP at positions distinct from that of PUT in the *Bv*HSS structure obtained from crystals after soaking with PUT (PDB ID: 4TVB, compare Fig. 6c with 6 a). Two DAP molecules were found at two different positions in subunit A (PDB ID: 4XQC), of which the first was found at the active site (Fig. 6c) and the second near the pore forming α-helix J in the entrance of the binding pocket (Fig. 8). The innermost nitrogen ND of the DAP at the active site is coordinated to Asn-162 and oxygen O7N of NAD^+^ , whereas nitrogen NAA is coordinated to Glu-237 and oxygen O2D of NAD^+^ . Neither nitrogen position exactly matches those of HSP or PUT. With the nitrogen NAA position being found near the inner amino site and the nitrogen ND position being found near the center amino site (shifted towards the inner amino site), the overall geometry of DAP has to be slightly “stretched” to fit the observed electron density. The innermost nitrogen ND of the second DAP is coordinated to Glu-117 and oxygen O2D of NAD^+^ , whereas nitrogen NAA is coordinated to Ser-236 and oxygen O of Glu-232 (Fig. 8). Thus, the nitrogen ND is positioned at the proposed ionic interaction site 2 of the “ionic slide” (refer to Fig. 2d). The other nitrogen is positioned slightly “below” the proposed ionic interaction site 1. In case of a PUT instead of a DAP, the corresponding nitrogen would still be positioned slightly below the ionic interaction site 1. This supports the proposed function of the ionic slide as being a substrate guiding mechanism: The observed electron density of this second DAP (Fig. 8) appears well defined and is not “smeared” as in the case of the first DAP. Thus, the modeled position is most likely homogeneously occupied throughout the most of protein molecules within the crystal. This positioning can be explained by a higher attractive force for a positively charged nitrogen of the ionic interaction site 2 over site 1, resulting in a substrate guiding towards the active site of *Bv*HSS.

### CAD and AGM were not Found at the Active Site of *Bv*HSS

We could not determine a distinct binding site for CAD. The electron density obtained from a *Bv*HSS crystal co-crystallized with CAD was not interpretable with bound CAD at the active site. All *Bv*HSS and *Bv*HSS variants co-crystallized with AGM were found to have AGM bound to the protein surface at various sites, most likely causing the observed improvement in crystal growth.

### *Bv*HSS Variants Exhibit Altered Substrate-Binding at the Active Site

Structures derived from the enzymatically inactive *Bv*HSS variant H296S (PDB ID: 4XQE and PDB ID: 4XRG) revealed slightly different substrate positions and no differences for any amino acid positions or orientations in the active site with the exception of Glu-237 and Glu-298. In contrast to wild-type *Bv*HSS, the variant H296S was found to bind AGM within the active site. The guanidine group of AGM was approximately placed at the former His-296 imidazole ring position (Fig. 6e). The primary amino group of AGM was bound and coordinated as that of HSP or PUT at the inner amino site. The electron density of *Bv*HSS variant H296S crystals co-crystallized with AGM and, when subsequently soaked for 5 min with PUT, showed a decrease in electron density for the guanidine group of the AGM bound at the active site. Therefore, we interpret the electron density as reflecting a partial replacement of AGM with PUT (Fig. 6f). Compared with PUT bound in wild-type *Bv*HSS (PDB ID: 4TVB), the first nitrogen of PUT is bound at the same inner amino site. The second nitrogen and the carbon that is thought to be oxidized occur approximately 1.5 Å farther away from the nicotine amide ring of NAD^+^ (approximately 2 Å and 1.5 Å, respectively, measured from the carbon C4N of NAD^+^ at which the hydride transfer occurs).

Enzymatically inactive *Bv*HSS variant E237Q co-crystallized with AGM and PUT resulted in a crystal structure with no bound substrate at the active site (PDB ID: 4XQG, Fig. 6d). Other than minimal and most likely negligible rotations of the side chain amide group of amino acid Gln-237 and the side chain of amino acid His-296, no structural changes of active site residues compared with wild-type *Bv*HSS were observed. In contrast to the structure of the wild-type *Bv*HSS with no bound substrate (PDB ID: 4PLP), the Tyr-123 was clearly represented by electron density in its narrow position. The exchange of residue Glu-237 by Gln-237 resulted in changes of the electrostatic potential of the surface of the binding pocket (Fig. 5g–i). Compared with wild-type *Bv*HSS, because of the change of the pK_a_ value the side chain of His-296 (pK_a,calc_ = 6.4) should no longer be protonated at both nitrogens resulting in a loss of the positive charge at the imidazole ring. Together with the loss of the negative charge of the side chain of residue 237 because of the replacement of glutamate by glutamine, the electrostatic potential of the surface of the pocket inverted at pH 7 and pH 9, at a region around Gln-237, to a now positive electrostatic potential and, at an area around His-296, to a negative electrostatic potential.

### Structures of Wild-type *Bv*HSS and *Bv*HSS Variants Provide Functional Insights

The structures of active wild-type *Bv*HSS and inactive *Bv*HSS variants reveal important amino acid residues for enzyme function and substrate binding. The side chain of His-296 seems to be crucial for correct substrate positioning. Of note, the structure of *Bv*HSS variant E237Q does not have PUT bound at the active site neither at the outer nor at the inner binding site. This implies an important role for the residue Glu-237 during substrate binding. Inverting the electrostatic potential around residue 237 upon Glu to Gln exchange will destroy the above-described model of the ionic slide und thus completely prevents the entry of PUT into the active site. In comparison with wild-type *Bv*HSS (PDB ID: 4PLP), for the E237Q variant the electron density of the residues of the “track-and-trace” loop is well defined. This indicates a rather rigid conformation of the “track-and-trace” loop and, with it, an extremely narrow and rigid opening of the pore of the binding pocket in the E237Q variant in relation to the wild-type *Bv*HSS. Under the assumption that the change of Glu-237 to Gln-237 mimics the electrostatic potential of a fully substrate bound state, the observed rigid and narrow conformation of the pore of the binding pocket supports the concept that it functions as a locking mechanism directed through a substrate-sensing feature of the “track-and-trace” loop.

### Hypothesis of the Reaction Steps of *Bv*HSS Catalysis

**Figure 9:**
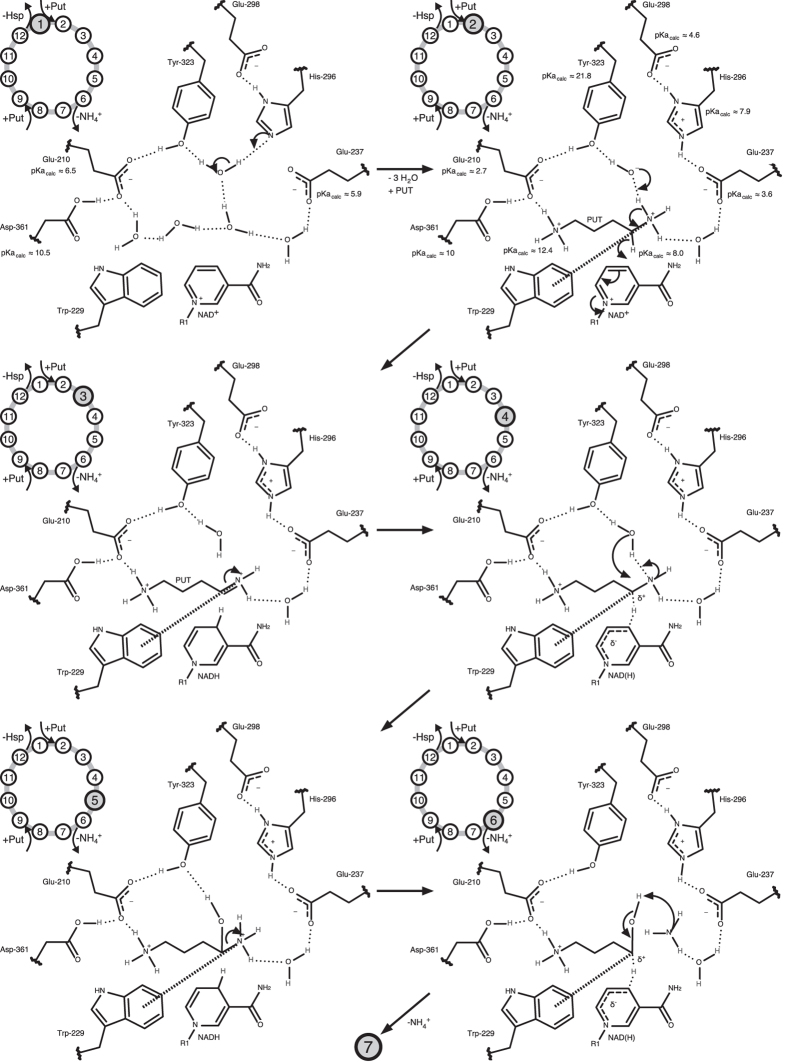
First six reaction steps of the bacterial HSS catalyzed formation of HSP from PUT. Relevant residues and NAD(H) of HSS, reaction substrates, intermediates, and products are shown as two-dimensional structure representations (see Fig. 3 for distances and orientation in three-dimensional space). The number of each step is indicated by the highlighted (gray background) number of the abstracted reaction circle in the upper left corner. The pK_a,calc_-values were calculated with the program PROPKA from the respective structures (step 1: *Bv*HSS with PDB ID: 4PLP, step 2: *Bv*HSS with PDB ID: 4TVB). The cation-π interaction of Trp-229 is indicated by a thick dashed line. Hydrogen bonds are indicated by thin dotted lines. Delocalized electrons/partial bonds are indicated by thin dashed lines.

**Figure 10:**
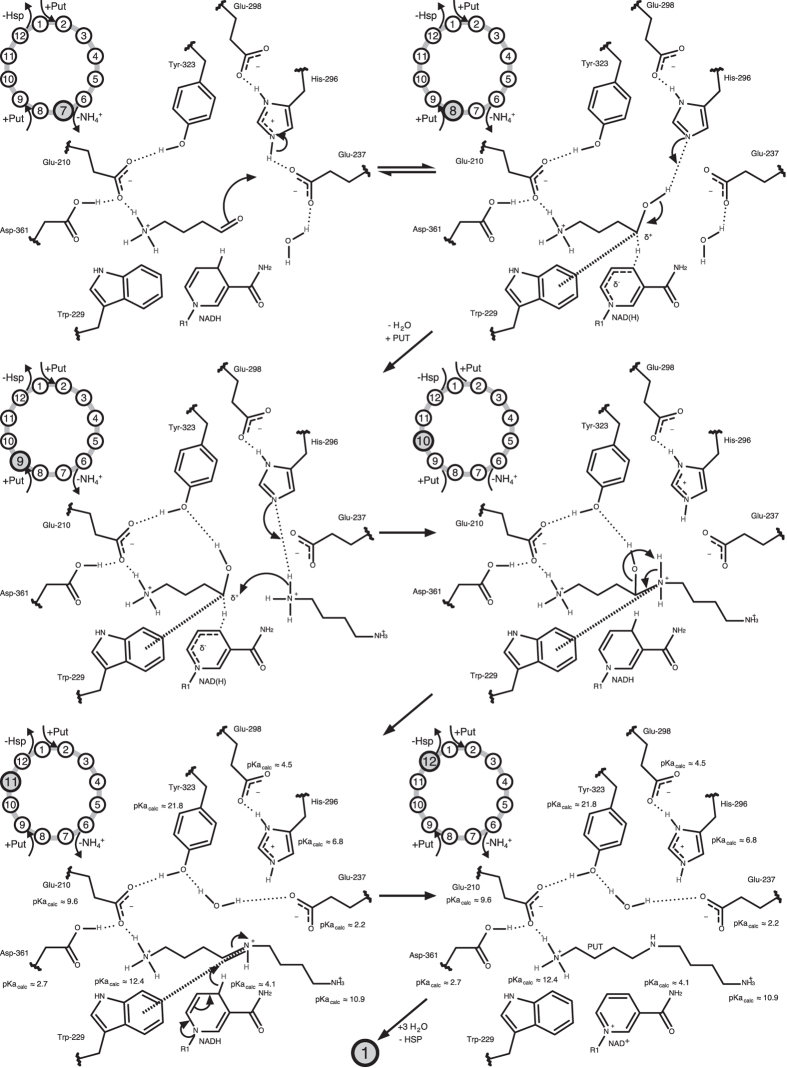
Last six reaction steps of the bacterial HSS catalyzed formation of HSP from PUT. Relevant residues and NAD(H) of HSS, reaction substrates, intermediates, and products are shown as two-dimensional structure representations (see Fig. 3 for distances and orientation in three-dimensional space). The number of each step is indicated by the highlighted (gray background) number of the abstracted reaction circle in the upper left corner. The pK_a,calc_-values were calculated with the program PROPKA from the respective structures (steps 11 and 12: *Bv*HSS with PDB ID: 4TVB). The cation-π interaction of Trp-229 is indicated by a thick dashed line. Hydrogen bonds are indicated by thin dotted lines. Delocalized electrons/partial bonds are indicated by thin dashed lines.

Based on the substrate-bound structures together with the calculated charge distribution in the binding pocket, we propose the following reaction steps for catalysis under neutral to basic conditions (Figs 9 and 10).

In combination, Glu-237 and Glu-298 transform His-296 into a strong base (calculated pK_a_ 10). This triad seems to enable the deprotonation of the nearby water shorty before or after binding of the first PUT molecule within the active site and thereby turning it into an active hydroxide ion (step 1, Fig. 9).

Upon the binding of the first PUT molecule at the inner site, repulsion between the positively charged His-296 and protonated amino group of PUT pushes PUT towards the nicotine amide ring of NAD^+^ , in favor of adopting an oxidation transition-state-like structure. The buildup of positive charge at the nitrogen of PUT at the central site might be stabilized via weak cation-π interaction with the neighboring Trp-229 (step 1 to 2, Fig. 9).

A proton transfer from the PUT nitrogen to the hydroxide ion initiates the hydride transfer from PUT carbon C4 to C4N of NAD^+^ leaving PUT with a protonated imine at C4 (step 2 to 3, Fig. 9).

In accordance with the observed electron density distribution of substrate bound *Bv*HSS, a hydride transition state with a partially aromatic nicotine amid ring and a positive partial charge at PUT carbon C4 seems probable. A near to optimal positioning of PUT carbon C4 (Fig. 7d) for cation-π interaction with Trp-229 makes it more likely that a positive partial charge occurs at PUT carbon C4 than at the PUT nitrogen (step 4, Fig. 9).

In this state, a proton transfer presumably occurs from water to the PUT nitrogen followed by a nucleophilic attack of the water/hydroxide ion oxygen at the electrophilic PUT carbon C4 (step 4 to 5, Fig. 9).

Because of the stabilization of the transient build up of a positive partial charge through cation-π stacking at PUT carbon C4, an ammonium ion most probably leaves, resulting in the complete hydrolysis of PUT to 4-aminobutanal (step 6 to 7, Figs 9 and 10).

This 4-aminobutanal might then be activated for nucleophilic attack at carbon C4 by protonation through His-296 and again a stabilization of a positive partial charge at its carbon by Trp-229 (step 7 to 8, Fig. 10).

After the entry of a second protonated PUT molecule into the active site, a proton transfer from PUT to His-296 could occur in concert with a nucleophilic attack by the nitrogen free electron pair at 4-aminobutanal carbon C4 (step 8 to 9, Fig. 10).

In contrast to the imine at the starting point of the reaction, the positive charge at nitrogen is in addition to the Trp-229 cation-π interaction stabilized by the second carbon chain making it more likely for the hydroxyl group to leave (step 10 to 11, Fig. 10).

The resulting protonated Schiff base will then be reduced to HSP by hydride re-transfer from NADH (step 11 to 12, Fig. 10), regenerating NAD^+^ .

Upon leaving the active site, the HSP might be protonated by His-296 at the secondary amine to restore the active site to its initial state (step 12 to 1, Fig. 10).

The proposed mechanism is in agreement with a previously postulated less detailed mechanism for HSS^8^,^9^,^34^. The HSS is able to utilize SPD as substrate to produce HSP and to produce (4-aminobutyl)(5-aminopentyl)amine, (4-aminobutyl)(6-aminohexyl)amine, or (4-aminobutyl)(7-aminheptyl)amine from PUT and respective diamines^9^. Additionally, HSS does not produce bis(3-aminopropyl)amine or bis(5-aminopentyl)amine from DAP or CAD^9^,^10^. In contrast to PUT, neither DAP nor CAD have been detected either at a distinct position at the inner binding site or at the outer binding site in HSS crystal structures. Instead, only DAP is found to swap potentially between the inner and outer sites without being oxidized. Thus, DAP can compete against PUT for both binding sites explaining its reported competitive inhibitory effect^9^. The aforementioned HSS products and the observed diamine binding patterns imply a highly specific PUT binding site at the inner site discriminating, via carbon chain length, at which position the redox reaction solely can take place. The outer binding site is probably less specific, and with the adjacent side pocket, it provides enough space for longer diamines. The observed lower conversion rates of diamines with longer carbon chains compared with PUT^9^ are most likely caused by the less favored binding of the increased hydrophobic parts inside the mainly negatively charged binding pocket. Additionally, the described “ionic slide” probably favors linear molecules with positive charges at approximately 6 Å distance.

All residues proposed to take part in substrate binding and the reaction catalysis (Asn-162, Glu-210, Trp-229, Glu-237, His-296, Glu-298, Tyr-323, and Asp-361) are highly conserved throughout the bacterial HSS family^13^ supporting observed substrate binding sites and the proposed mechanism. The non-functional *Bv*HSS variants E237Q, H296S, and E298Q, together with the structural arrangement of Glu-237, His-296, Glu-298, and water molecules (Figs 3 and 6) and the herein calculated pK_a_ value of approximately 10 for His-296, indicate a general base-catalyzed method of action comparable with that of the catalytic triad in serine protease with a nucleophilic water instead of a serine^35^. The first part of the bacterial HSS-catalyzed reaction can be described as an oxidative deamination, which in part resembles the reaction catalyzed by the structurally nonrelated NAD(P)^+^ -dependent glutamate dehydrogenase (GluDH, EC: 1.4.1.2-4). The reaction mechanism of GluDH from *Clostridium symbiosium* (*Cs*GluDH, PDB ID: 1BGV) has been analyzed in detail by Stillman *et al*.^36^ based on its 3D structure. A comparison of the two mechanisms reveals that the proposed deprotonation of the PUT nitrogen by a hydroxide ion is an initial driving force for oxidation at PUT carbon C4. Instead of an aspartate (Asp-165 in *Cs*GluDH), the *Bv*HSS utilizes most probably a well-shielded water/hydroxide ion as a proton acceptor to facilitate substrate oxidation and imine formation. As observed in substrate-bound *Bv*HSS (PDB ID: 4TVB), the Glu-237 carboxyl group at a distance of approximately 4.4 Å for each of its oxygens is much too far away from the PUT nitrogen to act as a base like Asp-165 in *Cs*GluDH. As in *Cs*GluDH, the deamination in *Bv*HSS occurs by a nucleophilic attack of a water molecule. The *Cs*GluDH initiates the nucleophilic attack by a proton transfer from Asp-165 to the imine nitrogen, thereby turning the imine carbon into a strong electrophile. The attacking water is activated by a proton transfer from a lysine (Lys-125)^36^. The *Bv*HSS lacks corresponding residues and seems to increase the electrophilic properties of PUT carbon C4 by stabilizing a positive partial charge at the carbon through cation-π interaction with Trp-229. This enables proton transfer from water to imine nitrogen followed by nucleophilic attack of the hydroxide ion at the imine carbon.

Cation-π interactions are described to be involved in the binding of cationic substrates^37^,^38^. For the cationic cyclization of squalene in steroid biosynthesis^39^,^40^,^41^, carbocation stabilization in retinal pigment epithelium-specific 65 kDa protein^42^, or eudesmane cation stabilization by Trp-334 of aristolochene synthase^43^, cation-π interactions are described to favor the reaction equilibrium towards product formation. The observed structural arrangements of *Bv*HSS Trp-229, PUT carbon C4, and PUT nitrogen N2 (PDB ID: 4TVB) similarly emphasize that cation-π interaction is involved in the stabilization of individual high-energy/transition states lowering energy barriers and thus favoring the reaction equilibrium towards product formation (compare Fig. 9 steps 3 to 6 and Fig. 10 steps 8 to 9).

The respective carbon (C4 and C05) of both substrates/intermediates found to bind at the active site of *Bv*HSS (PDB ID: 4TVB) are approximately positioned over the center of the 6-membered (benzene) ring of the Trp-229 side chain at a distance of 3.5 Å or 3.2 Å. The carbons lie in a near to optimal and much more favored position than the off-centered neighboring nitrogens of the respective molecule (see Fig. 7). This supports the hypothesis that, after oxidation and hydrolysis of PUT to 4-aminobutanal, a highly reactive state will be stabilized by cation-π interaction with Trp-229, thus supporting Schiff base formation with a second PUT.

Of note, the active site of the supposedly evolutionary related eukaryotic saccharopine dehydrogenase/reductase^13^ from *M. grisea* (*Mg*SacDH, PDB ID: 1E5Q^26^) has substrate, NADPH, and a tryptophan (Trp-174) arranged in a highly similar orientation as their equivalences (PUT/HSP, NAD^+^ , Trp-229) at the active site of *Bv*HSS. A key step of the proposed mechanism of the enzyme *Mg*SacDH is a Schiff base intermediate (between the substrates glutamate and α-aminoadipic-δ-semialdehyde), which is subsequently reduced by NADPH to saccharopine (forward reaction)^26^. Structure alignment of substrate-bound *Bv*HSS (PDB ID: 4TVB) and substrate-bound *Mg*SacDH (PDB ID: 1E5Q) reveals the tryptophan (Trp-229 of *Bv*HSS and Trp-174 of *Mg*SacDH) as the only conserved or converged amino acid in the vicinity of the active site. Although Johansson *et al*.^26^ did not consider the participation of tryptophan Trp-174 in enzyme catalysis, the described striking resemblances of *Bv*HSS and *Mg*SacDH with regard to their origin from evolutionary distant organisms support the proposed role of tryptophan Trp-229 during the enzyme catalysis of *Bv*HSS. In addition, the NAD(H)-dependent human DHS (PDB ID: 1RQD^29^), capable of producing HSP as a side reaction^44^, possesses some similar structural characteristics within its active site to those observed for *Bv*HSS. In addition to a histidine residue potentially functioning as general base^29^, a tryptophan (Trp-327) in DHS is oriented in a comparable mode towards the hydrid transfer site further supporting the proposed key function of tryptophan Trp-229 of *Bv*HSS. An enzyme-bound intermediate Schiff base within *Bv*HSS comparable to that observed for DHS^30^ is not possible because of the absence of a lysine residue at the active site of *Bv*HSS. This implies a distinct handling of the intermediate 4-aminobutanal between bacterial HSS and eukaryotic DHS. The potential presence of an intramolecular Schiff base as an intermediate forming 3,4-dihydro-2 H-pyrrole (five-membered ring) from 4-aminobutanal, as observed under polyamine extraction from *Bv*HSS^9^, seems to be unlikely to occur in its active site. Although the binding pocket theoretically provides enough space for a five-membered ring, the well-coordinated amino group at the inner amino-binding site, together with the linearly aligned carbon chain and the occurring ring strain, will prevent intramolecular in favor for intermolecular Schiff base formation. Thus, the 4-aminobutanal present at the inner binding site of the bacterial HSS will most likely immediately form a Schiff base with a diamine present at the outer binding site.

## Conclusion

Our study identifies important residues for substrate binding and enzyme function. It clearly supports the conclusion of Shaw *et al*.^13^ that the bacterial HSS is evolutionary not related to eukaryotic DHS/HSS but related to CA(N)SDH, lysine 6-dehydrogenase, saccharopine dehydrogenase, and aspartate dehydrogenase. In contrast to DHS, the active site is deeply buried inside the bacterial HSS monomer and provides no lysine for Schiff base formation. Nevertheless both enzymes have some comparable arrangements of amino acids at the active site: A Glu/Asp-His-Glu/Asp triad and a tryptophan near the hydride transfer site. Regarding the comparable reactions catalyzed by both enzymes, this converged motif strongly emphasizes the functional relevance of these residues. Thus, our study provides a solid base for follow up investigations of the proposed reaction mechanism.

With regard to HSS being considered as a drug target, our results lead to following implications: Because of the high specificity of the inner site for PUT, inhibitors targeting this site have to be highly similar to PUT. These inhibitors will bind not only to bacterial HSS, but most likely also to any enzyme utilizing or interacting with PUT, such as eukaryotic DHS and spermidine synthase^5^,^6^,^45^. Such a low drug/inhibitor specificity might cause severe cytotoxic site effects by interference with host polyamine metabolism^46^. Of note, our study has identified a side pocket (Fig. 2d) adjacent to the active site that might serve as a more specific inhibitor target, as it is not present in human DHS and is most probably an exclusive and conserved^13^ feature of bacterial HSS. An inhibitor binding in the side pocket could function by blocking substrate trafficking to the active site or it could reach partially into the active site occupying the outer substrate binding site. Additionally, the functionally relevant and highly conserved residue Glu-237 is accessible from the side pocket. Because of the observed flexible characteristics of the pore at the binding pocket entrance, even larger ring-shaped molecules might not be sterically hindered to enter the side pocket as long as they possess enough positive charges to outbalance their hydrophobicity.

## Materials and Methods

### Materials

DNA oligonucleotides/primers were obtained from Eurofins Genomics (Ebersberg, Germany); DNA-purification reagents and kits were obtained from Qiagen or Macherey-Nagel; if not stated otherwise, all other reagents were obtained from Sigma-Aldrich or Carl Roth.

### Generation of expression plasmids for HSS and HSS variants

The *Bv*HSS complementary DNA (cDNA) was amplified by touchdown polymerase chain reaction (PCR) with forward primer: 5′-ATA TAC CAT GGG AAC CGA TTG GCC GGT TTA TCA CCG CAT-3′ and reverse primer: 5′-ATA TAC TCG AGT CAG TCC CGC ACC AGC ACG TTG CGG AA-3′ by using a previously generated plasmid containing *Bv*HSS cDNA^34^ as template and *pfu* DNA polymerase (Promega) to introduce NcoI and XhoI restriction sites at the 5′ and 3′ ends, respectively. The PCR product was digested with NcoI and XhoI endonucleases (Promega). The *Bv*HSS cDNA was ligated into the NcoI/XhoI opened expression vector pETM14 (EMBL, providing an N-terminal 6xHis-tag followed by a human rhinovirus 3C (HRV3C) protease cleavage site). Ligation was performed with T4 DNA ligase (Invitrogen, now Life Technologies). The resulting plasmid pETM14-*Bv*HSS was transformed into the *E. coli* TOP10 strain for subsequent screening and control sequencing. Variants of *Bv*HSS were generated via side-directed mutagenesis according to the Quikchange protocol from Stratagene by using phusion high-fidelity DNA polymerase (Fermentas) or *pfu* DNA polymerase (Promega) with pETM14-*Bv*HSS plasmid as template and the following primers: HSS variant E237Q forward: 5′-GGT TTC GTG TCG CAG GGC CTG CAG C-3′, reverse: 5′-GCT GCA GGC CCT GCG ACA CGA AAC C-3′; HSS variant H296S forward: 5′-TAC GGC TTC CTG GTC ACC TCC AAC GAA TCG ATC TCG AT-3′, reverse: 5′-ATC GAG ATC GAT TCG TTG GAG GTG ACC AGG AAG CCG TA-3′; HSS variant E298Q forward: 5′-GGT CAC CCA CAA CCA ATC GAT CTC GAT C-3′, reverse: 5′-GAT CGA GAT CGA TTG GTT GTG GGT GAC C-3′; HSS variant N162D forward: 5′-GTG CTG CGG CGC CGA TCC CGG CAT GGT G-3′, reverse: 5′-CAC CAT GCC GGG ATC GGC GCC GCA GCA C-3′. Template plasmid was digested with restriction endonuclease DpnI prior to transformation of the reaction mixture into *E. coli* XL1 or TOP10 strains for subsequent screening and control sequencing. For *Bv*HSS expression, plasmids were transformed into the *E. coli* BL21 (DE3) expression strain.

### Expression and Purification of HSS

The BL21 (DE3) cells were cultivated in 1 L LB-Media (including 30 μg/ml kanamycine) in 5 l baffled Erlenmeyer flasks (at 37 °C in a standard laboratory shaking incubator) inoculated with an overnight culture to give a starting absorbance at 600 nm (OD_600_) of approximately 0.05. Induction occurred at an OD_600_ of approximately 0.6 with 1 mM isopropyl β-D-1-thiogalactopyranoside (IPTG) followed by a 4 h incubation at 25 °C. Cells were harvested by centrifugation (10 min, 7000 g, 4 °C) and either stored at −80 °C or kept on ice for immediate processing. Raw *E. coli* extract was prepared by disintegrating resuspended cells (1:10 (w:v) ratio in 50 mM BIS-TRIS propane pH 9, 25 mM KCl, 25 mM imidazole, 10 mM dithiothreitol (DTT), 1 mM phenylmethanesulfonyl fluoride (PMSF)) in an Avestin Emulsiflex C3 (1 passage using pulses of approximately 1200 bar) and two subsequent centrifugation steps (30 min, 75600 g, 4 °C). The *Bv*HSS was purified from the raw extract via immobilized metal ion affinity chromatography (IMAC) with the N-terminal 6xHis-tag by using a HisTrap HP 5 ml column and an ÄKTA Purifier FPLC system (both GE Healthcare). Protein was bound to the equilibrated Ni(II)-nitrilotri-acetic acid (NTA) matrix (10 column volumes [CV] of 50 mM BIS-TRIS propane pH 9, 25 mM KCl, 10 mM DTT [standard buffer]) followed by a washing step of 10 CV (25 mM imidazole in standard buffer) and a linear elution gradient over 5 CV to a final concentration of 500 mM imidazole in standard buffer. The *Bv*HSS eluted as a single, sharp, and symmetric peak at approximately 200 mM imidazole. To separate the 6xHis-tag from *Bv*HSS, the *Bv*HSS was incubated at 4 °C overnight with GST-tagged HRV3C protease (1:100 (w:w) ratio) in fresh standard buffer. Buffer was exchanged according to the manufacturer’s recommended standard procedure with HiTrap Desalting columns (GE Healthcare). The HRV3C protease and the 6xHis-tag were removed by coupled GSTrap and HisTrap columns. Purity of the HSS was verified by analytical size-exclusion chromatography (Superdex 75, 10/30HR, GE Healthcare) and photon correlation spectroscopy/dynamic light scattering (Zetasizer Nano-S, Malvern). HSS was stored in aliquots at a concentration between 5–6 mg/ml in standard buffer supplemented with 2 mM NAD^+^ at −80 °C.

### HSS activity assay

*Bv*HSS activity was tested according to a modified protocol published in Ober *et al*.^9^. Briefly, enzyme assays were performed in a total volume of 25 μl in 100 mM glycine NaOH buffer pH 9.0 containing 1 mM [1,4-^14^C]-putrescine (0.025 μCi/assay) and 2 mM NAD^+^ . Assays were incubated for 2 to 8 min at 37 °C. Formation of HSP was followed quantitatively by thin layer chromatography equipped with a radio-scanner (RITA, Raytest, Straubenhardt, Germany).

### Qualitative HSS activity assay for HPLC-based polyamine analysis

The enzyme (0.5 mg/ml) was incubated at 37 °C for 1 h with 2 mM PUT in standard buffer supplemented with 2 mM NAD^+^ in a total volume of 100 μl. The reaction was stopped by precipitation with trichloroacetic acid and analyzed by high-pressure liquid chromatography (HPLC; sample preparation according to the preparation of samples from *Bv*HSS crystals as described under section “Sample preparation for HPLC analysis”).

### Sample preparation for HPLC analysis

For polyamine content analysis of *Bv*HSS crystals, the soaking of crystals obtained from the co-crystallization of *Bv*HSS with DAP was performed with PUT for 300 s as described under crystallization and data collection. Samples from *Bv*HSS crystals were prepared by transferring single or multiple crystals with minimal amounts of mother liquor from the drop of the crystallization or soaking solution into a 0.5 μl drop of 0.2 M borate buffer pH 8.5 followed by immediate protein precipitation via addition of 0.5 μl 0.6 M trichloroacetic acid. Precipitated protein was removed by centrifugation for 1 min at 16000 g. The supernatant was transferred to a new tube and brought to approximately pH 8.5 with a final volume of 10 μl by addition of 9 μl 0.2 M borate buffer pH 8.5.

Each sample was derivatized for 5 min in the dark at room temperature by addition of a 10 mM 6-aminoquinolyl-N-hydroxysuccinimidyl carbamate (Synchem UG & Co. KG, Altenburg, Germany) solution in anhydrous acetonitrile at a 1:2.5 (v:v) ratio followed by immediate analysis.

### HPLC-based polyamine analysis

Polyamine composition was analyzed according to Weiss *et al*.^47^. Separations were performed on a C18 column (250 × 4.6 mm, ProntoSIL Hypersorb ODS, F180PY050, Bischoff) by using an Agilent 1100 Series HPLC system (Degasser G1379A, Quat Pump G1311A, Man. Inj. G1328B, COLCOM G1316A, DAD G1315B, FLD G1321A, RID G1362A, Agilent Technologies) controlled by ChemStation software version B.01.03 (Agilent Technologies).

The mobile phase consisted of a gradient (details see Weiss *et al*.^47^) of solvent A (25 mM triethylamine, titrated to pH 4.8 with acetic acid), solvent B (acetonitrile:water 80:20 (v/v)), and solvent C (methanol). Samples of 10 μl were injected into the column and analyzed at a flow rate of 1.3 ml/min at 33 °C with online UV detection at 210 nm and online fluorescence detection at λ_Ex_ = 248 nm, λ_Em_ = 398 nm.

Retention times were verified by analysis of calibration mixtures containing approximately 0.8, 8, or 80 pmol DAP, PUT, SPD, and HSP at appropriate time points during the analysis. The general applicability of sample preparation and polyamine content analysis of samples containing *Bv*HSS was verified by performing qualitative HSS activity assays followed by HPLC-based analyses.

### Crystallization and data collection

*Bv*HSS without additional polyamines could be crystallized at 18 °C by the hanging-drop vapor-diffusion method by using drops consisting of 1 μl protein solution (4 mg/ml in standard buffer) and 1 μl reservoir solution equilibrated against 500 μl reservoir solution (100 mM sodium acetate pH 4.6, 100 mM ammonium acetate, 34% (w/v) PEG 3350). *Bv*HSS and *Bv*HSS variants with polyamines could be crystallized at 18 °C by the hanging-drop vapor-diffusion method by using drops consisting of 1 μl protein solution (4 mg/ml in 33 mM BIS-TRIS propane pH 9, 17 mM KCl, 6.7 mM DTT, 1.3 mM NAD) including 0.2 M of each polyamine (1:2650 molecular ratio to guarantee substrate saturation, see Table 3 for details) and 1 μl reservoir solution equilibrated against 500 μl reservoir solution (100 mM sodium acetate pH 4.6–4.8, 150 mM ammonium acetate, 22–26% (w/v) PEG 10000, 150–300 mM 3-pyridin-1-ium-1-ylpropane-1-sulfonate [NDSB-201]). Crystals appeared after 3-5 d. In contrast to *Bv*HSS without polyamines growing as single crystals, the *Bv*HSS and *Bv*HSS variants with polyamines exclusively led to rod-shaped crystal clusters. Clustered crystals were carefully manually disintegrated to obtain single rod-shaped crystals. In the case of *Bv*HSS without additional polyamines, the crystals were cryoprotected by brief equilibration in 60% (v/v) reservoir solution in double-distilled H_2_O (ddH_2_O) supplemented with 10% (v/v) PEG 400 prior to flash-cooling in liquid N_2_. The soaking of *Bv*HSS crystals with polyamines was performed by transferring crystals into a 2:3 dilution of the respective reservoir solution with ddH_2_O (67 mM sodium acetate pH 4.6–4.8, 100 mM ammonium acetate, 14.7–17.3% (v/v) PEG 10000, 100–200 mM NDSB-201) containing 0.2 M polyamine and incubated for 5 min at 18 °C prior to cooling. *Bv*HSS and *Bv*HSS variants with polyamines were flash-cooled in liquid N_2_ without any cryoprotectant.

Diffraction data were collected at beam line 14.2 (Joint Berlin MX Lab, BESSY II, Berlin-Adlershof, Germany) by using a RAYONIX MX-225 CCD detector (structure with PDB ID: 4PLP, wavelength 0.918 Å), beam lines P13 and P14 (EMBL, DESY PETRA III, Hamburg, Germany) by using a Pilatus 6 M-F detector (structures with PDB ID: 4TVB, wavelength 1.23953 Å , PDB ID: 4XR4, wavelength 1.23953 Å, PDB ID: 4XQC, wavelength 0.976261 Å, PDB ID: 4XQE, wavelength 0.9763 Å, PDB ID: 4XRG, wavelength 0.9763 Å, and PDB ID: 4XQG, wavelength 1.03322 Å) and beam line ID23 (ESRF, Grenoble, France) by using a ADSC Quantum Q315R detector (structure with PDB ID: 4XQ9, wavelength 0.97628 Å). X-ray diffraction was performed at a temperature of 100 K.

### Structure determination, interpretation, and representation

Diffraction data were indexed and integrated by using the software XDS^48^. The space group was determined with the program Pointless of the CCP4 program suite^49^,^50^. Data were scaled and merged together by applying the FreeR-flag to 5% of reflections by using SCALA software of the CCP4 program suite^49^,^50^. The structure of *Bv*HSS without polyamines (PDB ID: 4PLP) was solved via molecular replacement (MR; resolution range of used data: 47.59–3.0 Å; resulting correlation coefficient: 0.312 [correct solution], 0.186 [second unrelated peak]; resulting R_factor_: 0.527 [correct solution], 0.570 [second unrelated peak]) by using the software MOLREP within the CCP4 program suite^50^,^51^,^52^. The starting model for MR was built by the PHYRE server by using the HSS structure from *L. pneumophila* (PDB ID: 2PH5, subunit A, 44% sequence identity)^53^. MR was followed by *Bv*HSS automated model building with the software ARP/wARP^54^. The *Bv*HSS model was iteratively completed by alternating refinement steps with the software PHENIX.refine^55^ by using all collected data as deposited in the PDB. The model to data fit was verified and improved by manual inspection and modification by using the program Coot^56^ and the CC_1/2_/CC^*^/CC_work_/CC_free_ coefficients^57^. The refined model (PDB ID: 4PLP) was used as starting model for structure determination from all other data sets collected from crystals of *Bv*HSS and *Bv*HSS variants by using the same procedure as described above. The Ramachandran statistics were (favored [%], allowed [%], outliers [%]): PDB ID: 4PLP (97, 2.9, 0.1), PDB ID: 4TVB (97.3, 2.7, 0), PDB ID: 4XR4 (97.4, 2.6, 0), PDB ID: 4XQ9 (97.4, 2.6, 0), PDB ID: 4XQC (97.4, 2.6, 0), PDB ID: 4XQE (97.4, 2.6, 0), PDB ID: 4XRG (97.0, 3.0, 0), PDB ID: 4XQG (97.6, 2.4, 0).

The pH-dependent electrostatic potential maps were calculated by using the software APBS Version 1.3^58^ with the AMBER99 force field^59^. Input files in PQR-format for APBS were generated from files containing *Bv*HSS atom coordinates in PDB-format with a modified version of the program PDB2PQR version 1.9.0 (to consider ligands in the pK_a_ prediction, PROPKA version 3.0 was replaced with version 3.1) in order to calculate pK_a_ values and set protonation states for titratable groups accordingly^60^,^61^. The volume and surface of the binding pocket was calculated with the software HOLLOW^28^ by filling the interior of the protein with dummy atoms (1.4 Å radius) on a grid (spacing 0.2 Å) and manually including water molecules based on the structure from *Bv*HSS (PDB ID: 4PLP).

All visualization and preparation of 3D structural images was performed with PyMOL^62^.

## Additional Information

**Accession Codes:** The atomic coordinates and structure factors (codes PDB ID: 4PLP, PDB ID: 4TVB, PDB ID: 4XR4, PDB ID: 4XQ9, PDB ID: 4XQC, PDB ID: 4XQE, PDB ID: 4XRG and PDB ID: 4XQG) have been deposited in the Protein Data Bank (http://wwpdb.org/).The atomic coordinates used for initial model building for molecular replacement (code PDB ID: 2PH5) and those referenced in the results and discussion sections [codes PDB ID: 4INA, PDB ID: 2AXQ25, PDB ID: 1E5Q26, PDB ID: 1BGV36 and PDB ID: 1RQD29] can be found in the Protein Data Bank (http://wwpdb.org/).

**How to cite this article**: Krossa, S. *et al*. Comprehensive Structural Characterization of the Bacterial Homospermidine Synthase–an Essential Enzyme of the Polyamine Metabolism. *Sci. Rep.* **6**, 19501; doi: 10.1038/srep19501 (2016).

## Supplementary Material

Supplementary Information

## Figures and Tables

**Figure 1:**
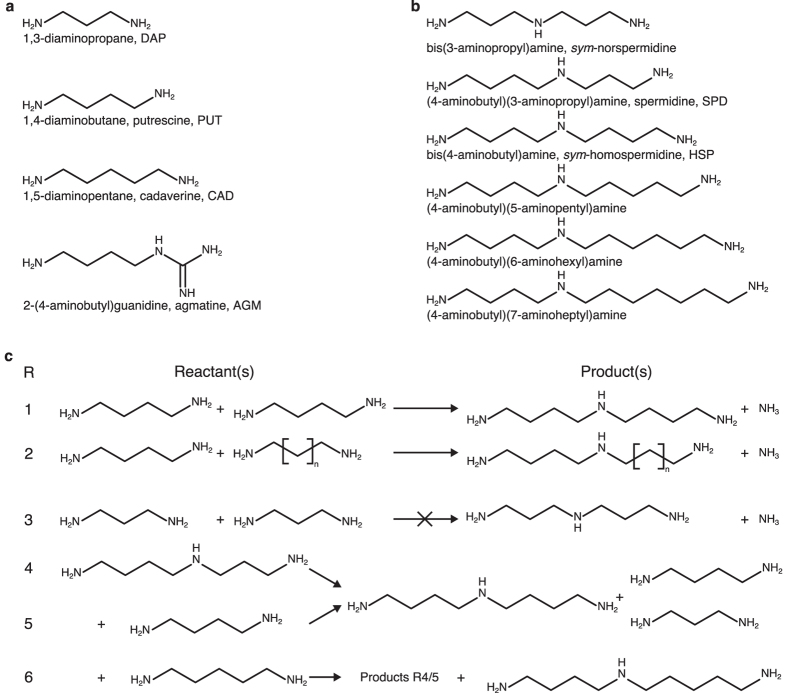
Overview of bacterial HSS-related polyamines and bacterial HSS-catalyzed reactions. (**a**) Two-dimensional structures of diamines (DAP, PUT, CAD) and agmatine (AGM). (**b**) Two-dimensional structures of triamines. (**c**) Known net reactions of the bacterial HSS^9^: Reaction 1: HSP formation from 2× PUT; reaction 2: general triamine formation from PUT and a second diamine (with n = [1 to 5]); reaction 3: bacterial HSS does not produce *sym*-norspermidine from 2× DAP; reaction 4: HSP, DAP, and PUT formation from SPD; reaction 5: HSP, DAP, and PUT formation from SPD and PUT; reaction 6: (4-aminobutyl)(5-aminopentyl)amine, HSP, DAP, and PUT formation from SPD and CAD.

**Table 1:** Data collection and refinement statistics*.

	*Bv*HSS w/o substrate; PDB ID: 4PLP	*Bv*HSS with DAP (co-cryst.), PUT (soak); PDB ID: 4TVB	*Bv*HSS with AGM (co-cryst.); PDB ID: 4XR4	*Bv*HSS with CAD (co-cryst.); PDB ID: 4XQ9	*Bv*HSS with DAP, PUT (co-cryst.); PDB ID: 4XQC	*Bv*HSS H296S with AGM (co-cryst.); PDB ID: 4XQE	*Bv*HSS H296S with AGM (co-cryst.), PUT (soak); PDB ID: 4XRG	*Bv*HSS E237Q with AGM, PUT (co-cryst.); PDB ID: 4XQG
Data collection								
Space group	P2_1_2_1_2_1_	P22_1_2_1_	P22_1_2_1_	P22_1_2_1_	P22_1_2_1_	P22_1_2_1_	P22_1_2_1_	P22_1_2_1_
Cell dimensions								
*a, b, c* (Å)	70.1, 109.8, 193.0	59.5, 109.3, 157.2	54.9, 108.6, 161.3	60.1, 110.7, 157.5	60.1, 109.8, 157.3	60.3, 110.1, 157.9	60.0, 110.2, 157.5	59.8, 109.3, 157.6
α β γ (°)	90, 90, 90	90, 90, 90	90, 90, 90	90, 90, 90	90, 90, 90	90, 90, 90	90, 90, 90	90, 90, 90
Resolution (Å)	47.71–1.8 (1.86–1.8)*	89.72–1.9 (1.97–1.9)	90.06–1.65 (1.71–1.65)	47.75–1.9 (1.97–1.9)	63.95–1.5 (1.55–1.5)	90.30–1.6 (1.66-1.6)	90.31–1.6 (1.66-1.6)	89.79–1.5 (1.55–1.5)
*R*_merge_	0.123 (0.858)	0.197 (1.153)	0.088 (0.754)	0.199 (1.036)	0.103 (0.630)	0.100 (0.743)	0.107 (0.757)	0.088 (0.701)
*I*/σ*(I)*	9.4 (2.0)	11.4 (2.3)	14.4 (2.1)	9.2 (2.0)	11.6 (2.7)	12.6 (2.6)	10.6 (2.1)	12.4 (2.1)
Completeness (%)	99.16 (99.44)	99.97 (100)	99.86 (99.32)	99.98 (99.95)	97.33 (96.13)	99.68 (99.22)	99.82 (99.63)	98.65 (98.55)
Redundancy	3.7 (3.7)	13.3 (13.2)	6.4 (5.2)	7.1 (5.5)	6.9 (6.9)	6.6 (6.6)	6.0 (5.1)	6.7 (6.8)
Refinement								
Resolution (Å)	47.71–1.8 (1.86–1.8)*	89.72–1.9 (1.97–1.9)	90.06–1.65 (1.71–1.65)	47.75–1.9 (1.97–1.9)	63.95–1.5 (1.55–1.5)	90.30–1.6 (1.66-1.6)	90.31–1.6 (1.66-1.6)	89.79–1.5 (1.55–1.5)
No. of unique reflections	137095 (13566)	81583 (8063)	116495 (11465)	83517 (8244)	162300 (15818)	138541 (13623)	138081 (13615)	163288 (16076)
*R*_work_/*R*_free_	0.170/0.205 (0.306/0.340)	0.168/0.195 (0.235/0.254)	0.136/0.172 (0.221/0.264)	0.141/0.184 (0.227/0.263)	0.136/0.164 (0.178/0.211)	0.128/0.165 (0.179/0.238)	0.140/0.175 (0.199/0.249)	0.126/0.156 (0.179/0.224)
No. atoms								
Protein	7573	7457	7504	7503	7563	7653	7549	7537
Ligand/ion	92	118	219	96	166	217	165	219
Water	1035	1298	984	1307	1490	1086	1155	1146
*B*-factors								
Protein	27.9	20.4	19.7	22.3	13.8	19.3	21.4	17.7
Ligand/ion	21.8	16.7	29.5	20.2	16.7	24	23.4	25.1
Water	42.3	29.8	34	36.6	27.3	34.2	36.1	32.1
R.m.s. deviations								
Bond lengths (Å)	0.012	0.01	0.020	0.008	0.01	0.012	0.009	0.012
Bond angles (°)	1.41	1.17	1.34	1.15	1.31	1.38	1.26	1.36

^*^Each structure is based on data collected from one crystal. Values in parenthesis are for highest resolution shell.

**Table 2:** Structure alignment of *Bv*HSS subunit A to subunit B:

	Subunit B to A [Å^2^] (residues 3-476 , super)	Subunit B to A [Å^2^] (residues 3-476 , rms_cur)	“Track-and-trace” loop, subunit B to A [Å^2^] (residues 120-130, rms_cur)	Subunit B to A [Å^2^] (NAD(H), rms_cur)
*Bv*HSS w/o substrate; PDB ID: 4PLP	0.15 (2786 atoms)	0.81 (3699 atoms)	1.72 (93 atoms)	0.58 (44 atoms)
*Bv*HSS with DAP (co-cryst.), PUT (soak); PDB ID: 4TVB	0.13 (2853 atoms)	0.68 (3699 atoms)	0.57 (93 atoms)	0.57 (44 atoms)

Aligned with the “super” algorithm as implemented in the program PyMOL (5 cycles) to align subunit B residues 3-476 (complete residue without hydrogens, always using alternate location A) to chain A of respective *Bv*HSS structure; all other RMSD values were calculated with the rms_cur function as implemented in the program PyMOL for complete residues without hydrogens, always using alternate location A. The respective residue range and the used alignment method are given in round parenthesis in the first row of the table. The number of atoms of the aligned parts of both molecules used to calculate each RMSD is given in parenthesis.

**Table 3:** Crystallization conditions and polyamine composition of *Bv*HSS crystals.

	Polyamine(s) in protein solution	Polyamine soaked	Polyamine identified in active site	Reservoir pH	Reservoir PEG (w/v)	Reservoir NDSB-201
*Bv*HSS w/o substrate; PDB ID: 4PLP	—	—	—	4.6	34% PEG 3350	—
*Bv*HSS with DAP (co-cryst.), PUT (soak); PDB ID: 4TVB	0.2 M DAP	300 s 0.2 M PUT	HSP, PUT (transition close states)	4.8	22% PEG 10000	250 mM
*Bv*HSS with AGM (co-cryst.); PDB ID: 4XR4	0.2 M AGM	—	—	4.8	22% PEG 10000	150 mM
*Bv*HSS with CAD (co-cryst.); PDB ID: 4XQ9	0.2 M CAD	—	—	4.8	22% PEG 10000	150 mM
*Bv*HSS with DAP, PUT (co-cryst.); PDB ID: 4XQE	0.2 M DAP, 0.2 M PUT	—	DAP	4.6	24% PEG 10000	300 mM
*Bv*HSS H296S with AGM (co-cryst.); PDB ID: 4XQE	0.2 M AGM	—	AGM	4.6	22% PEG 10000	250 mM
*Bv*HSS H296S with AGM (co-cryst.), PUT (soak); PDB ID: 4XRG	0.2 M AGM	300 s 0.2 M PUT	AGM+PUT	4.6	24% PEG 10000	300 mM
*Bv*HSS E237Q with AGM, PUT (co-cryst.); PDB ID: 4XQG	0.2 M AGM, 0.2 M PUT	—	—	4.6	24% PEG 10000	300 mM

Reservoir solution consisted of 100 mM sodium acetate buffer pH 4.6–4.8 and 100 mM (*Bv*HSS w/o substrate; PDB ID: 4PLP) or 150 mM (all other crystals) ammonium acetate in addition to items listed in the Table. Protein solution consisted of approximately 4 mg/ml *Bv*HSS in standard buffer (*Bv*HSS w/o substrate; PDB ID: 4PLP) or in 33 mM BIS-TRIS propane pH 9, 17 mM KCl, 6.7 mM DTT, 1.3 mM NAD (all other crystals).

**Table 4:** Alignment of all *Bv*HSS structures to *Bv*HSS structure with the PDB ID: 4PLP:

	Subunit A [Å^2^] (residues 3-476 , super)	Subunit A to subunit A of 4PLP [Å^2^] (residues 3-476 , rms_cur)	Subunit B to subunit B of 4PLP [Å^2^] (residues 3-476 , rms_cur)	“Track-and-trace” loop, subunit A [Å^2^] (residues 120-130, rms_cur)	Loop A, subunit A [Å^2^] (residues 181-188, rms_cur)	Loop B, subunit A [Å^2^] (residues 453-465, rms_cur)
*Bv*HSS with DAP (co-cryst.), PUT (soak); PDB ID: 4TVB	0.21 (2951 atoms)	0.80 (3699 atoms)	1.51 (3699 atoms)	2.88 (93 atoms)	1.45 (55 atoms)	0.78 (98 atoms)
*Bv*HSS with AGM (co-cryst.); PDB ID: 4XR4	0.23 (3122 atoms)	0.78 (3691 atoms)	1.13 (3691 atoms)	2.81 (93 atoms)	1.76 (55 atoms)	0.46 (98 atoms)
*Bv*HSS with CAD (co-cryst.); PDB ID: 4XQ9	0.18 (2990 atoms)	0.66 (3699 atoms)	1.44 (3699 atoms)	1.67 (93 atoms)	1.48 (55 atoms)	0.81 (98 atoms)
*Bv*HSS with DAP, PUT (co-cryst.); PDB ID: 4XQC	0.21 (3068 atoms)	0.69 (3699 atoms)	1.48 (3699 atoms)	1.72 (93 atoms)	1.68 (55 atoms)	0.90 (98 atoms)
*Bv*HSS H296S with AGM (co-cryst.); PDB ID: 4XQE	0.22 (3035 atoms)	0.73 (3689 atoms)	1.49 (3689 atoms)	1.42 (93 atoms)	1.70 (55 atoms)	0.80 (98 atoms)
*Bv*HSS H296S with AGM (co-cryst.), PUT (soak); PDB ID: 4XRG	0.22 (3125 atoms)	0.71 (3689 atoms)	1.04 (3689 atoms)	1.24 (93 atoms)	1.90 (55 atoms)	0.62 (98 atoms)
*Bv*HSS E237Q with AGM, PUT (co-cryst.); PDB ID: 4XQG	0.22 (2988 atoms)	0.73 (3690 atoms)	1.59 (3690 atoms)	1.72 (93 atoms)	1.55 (55 atoms)	0.87 (98 atoms)

Aligned with the “super” algorithm as implemented in the program PyMOL (5 cycles) to align subunit A residues 3-476 (complete residue without hydrogens, always using alternate location A) to 4PLP subunit A; all other RMSD values were subsequently calculated for the superimposed proteins with rms_cur function implemented in the program PyMOL for complete residues without hydrogens, always using alternate location A. The respective residue range and the used alignment method are given in round parenthesis in the first row of the table. The number of atoms of the aligned parts of both molecules used to calculate each RMSD is given in parenthesis.
