# Supplementary material for: Comprehensive Structural Characterization of the Bacterial Homospermidine Synthase–an Essential Enzyme of the Polyamine Metabolism
Source: Sci Rep. 2016 Jan 18;6:19501. doi: 10.1038/srep19501 (PMC4725965; doi:10.1038/srep19501)
Supplement: Supplementary Information [file srep19501-s1.pdf]

Supplemental Information to

Comprehensive structural characterization of the bacterial Homospermidine Synthase – an essential enzyme of the polyamine metabolism.

Sebastian Krossa[‡1], Annette Faust [‡1], Dietrich Ober[§] & Axel J. Scheidig[‡2]

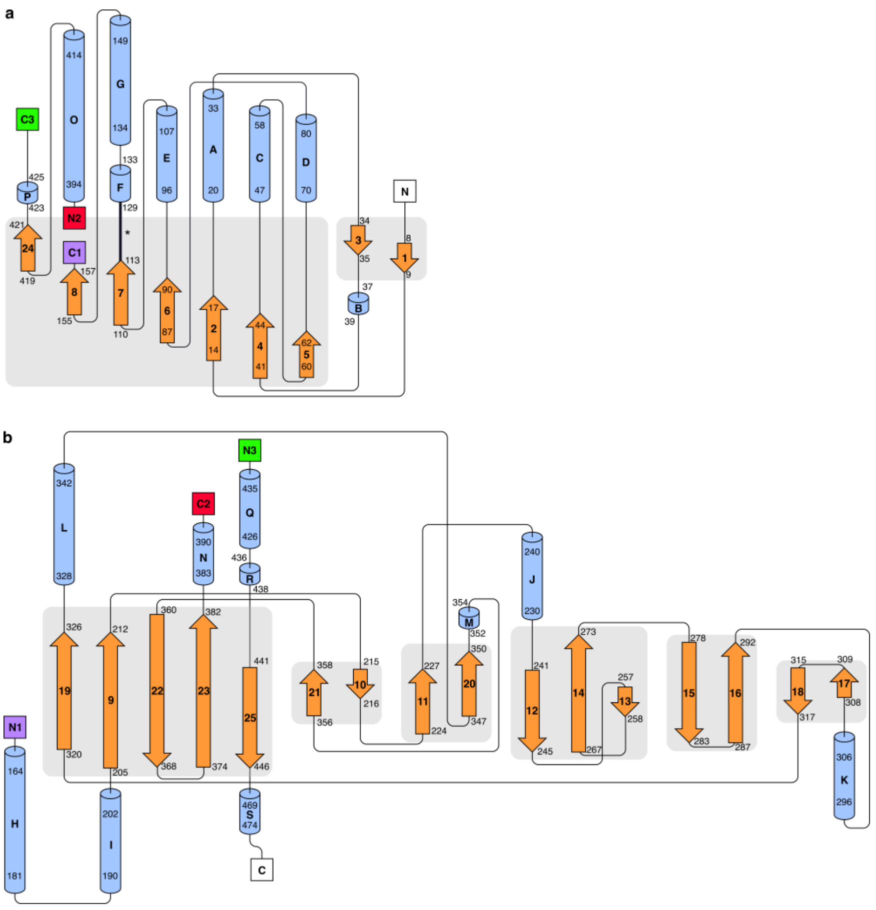

Supplementary Figure S1: Topology diagram of *Bv*HSS. The diagram was generated with the program Pro-origami[1] by using secondary structure information embedded into the PDB file for *Bv*HSS subchain A (PDB ID: 4PLP). α-Helixes (numbered alphabetically A to S) are shown as cylinders, and β-strands (numbered 1 to 25) are shown as arrows. The numbers of the first and last residue of every secondary

structure element are given at the start and end point of each representation. β-Sheets are indicated as boxes. (a) Domain 1 – "NAD(P)-binding Rossmann-like" domain. The "track-and-trace" loop is represented by a thick black line and indicated by an asterisk. (b) Domain 2 – "homospermidine-synthase–like" domain.

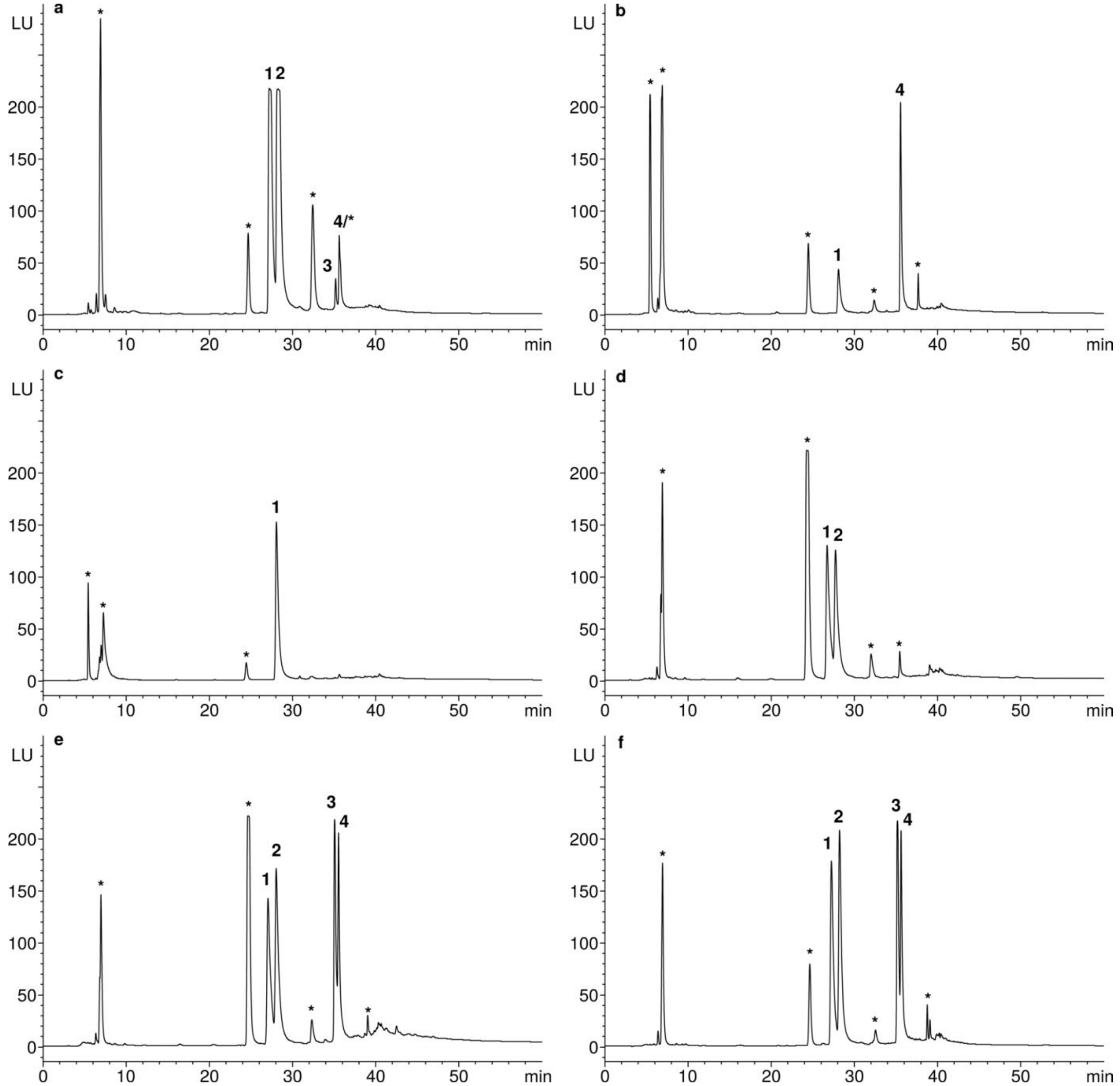

Supplementary Figure S2: Results of HPLC-based polyamine analyses of *Bv*HSS crystals, qualitative enzyme activity assays, and various calibration mixtures. The chromatograms show the measured fluorescence intensity in light units (LU) (detector-settings: λ_{Ex} = 248 nm; λ_{Em} = 398 nm; photomultiplier

tube gain 11; response time 4 s (standard); lamp flash frequency 296 Hz (standard)) over the complete analysis run time of 60 min. (a) Analysis of the polyamine content of a *Bv*HSS crystal cluster from co-crystallization with DAP and PUT (sample A). (b) Analysis of polyamine content after incubation of *Bv*HSS with PUT in standard buffer supplemented with 2 mM NAD^+ for 1 h at 37 °C. (c) Analysis of polyamine content after incubation of non-functional *Bv*HSS variant H296S with PUT in standard buffer supplemented with 2 mM NAD^+ for 1 h at 37 °C. (d) Chromatogram of calibration mixture I, containing DAP and PUT each with approximately 800 pmol. (e, f) Chromatograms of two independent separations of calibration mixtures II, containing DAP, PUT, SPD, and HSP, each with approximately 800 pmol. Polyamines identified via retention times: 1 = DAP, 2 = PUT, 3 = SPD, 4 = HSP. Impurities that were not further analyzed or byproducts of sample derivatization are marked with an asterisk (*).

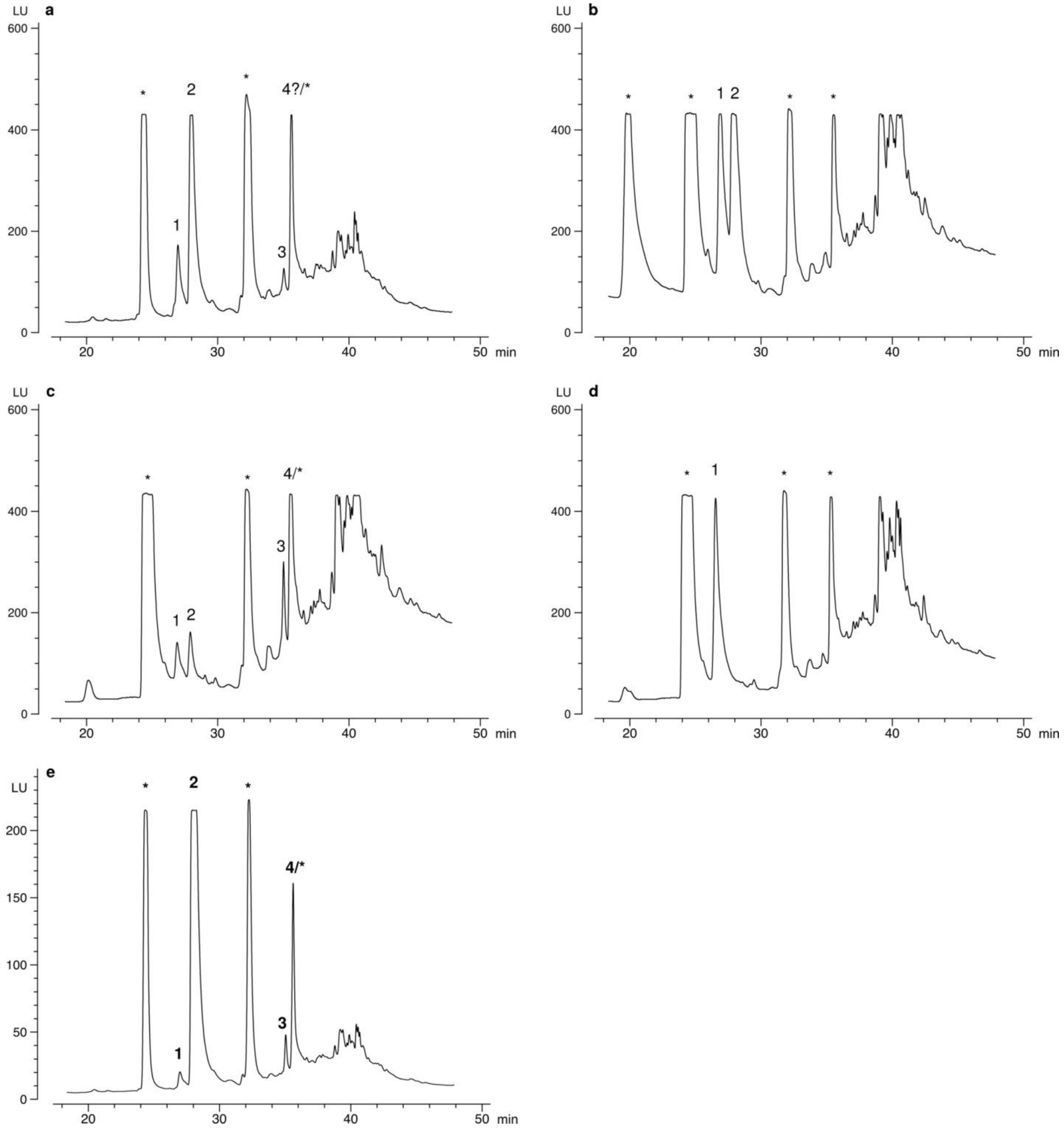

Supplementary Figure S3: Results of HPLC-based polyamine analyses of *Bv*HSS crystals and various calibration mixtures. The chromatograms show the measured fluorescence intensity in light units (LU) (detector-settings: λ_{Ex} = 248 nm; λ_{Em} = 398 nm; photomultiplier tube gain 16 (a-d) or 14 (e); response time 4 s (standard); lamp flash frequency 296 Hz (standard)) over the complete analysis run time of 60 min. (a) Analysis of the polyamine content of a single *Bv*HSS crystal from co-crystallization with DAP

and subsequent soaking with PUT (sample C). (b) Chromatogram of calibration mixture I, containing DAP and PUT, each with approximately 8 pmol. (c) Chromatogram of calibration mixtures II, containing DAP, PUT, SPD, and HSP, each with approximately 0.8 pmol. (d) Chromatogram of calibration mixture III, containing PUT with approximately 8 pmol. (e) Analysis of the polyamine content of a *Bv*HSS crystal cluster from co-crystallization with DAP and subsequent soaking with PUT (sample B). Polyamines identified via retention times: 1 = DAP, 2 = PUT, 3 = SPD, 4 = HSP. Impurities that were not further analyzed or byproducts of sample derivatization are marked with an asterisk (*).

References

1 Stivala, A., Wybrow, M., Wirth, A., Whisstock, J. C. & Stuckey, P. J. Automatic generation of protein structure cartoons with Pro-origami. *Bioinformatics* **27**, 3315-3316, doi:10.1093/bioinformatics/btr575 (2011).
